# Subsistence of early anatomically modern humans in Europe as evidenced in the Protoaurignacian occupations of Fumane Cave, Italy

**DOI:** 10.1038/s41598-023-30059-3

**Published:** 2023-03-07

**Authors:** Ana B. Marín-Arroyo, Gabriele Terlato, Marco Vidal-Cordasco, Marco Peresani

**Affiliations:** 1 Grupo de I+D+I EVOADAPTA (Evolución Humana y Adaptaciones durante la Prehistoria), Dpto. Ciencias Históricas, Universidad de Cantabria, Avda. de Los Castros 44, 39005 Santander, Spain; 2 Dipartimento di Studi Umanistici, Sezione di Scienze Preistoriche e Antropologiche, Università di Ferrara, Ferrara, Italy; 3 Istituto di Geologia Ambientale e Geoingegneria, Consiglio Nazionale delle Ricerche, Milan, Italy

**Keywords:** Ecological modelling, Archaeology, Cultural evolution

## Abstract

Documenting the subsistence strategies developed by early modern humans is relevant for understanding the success of their dispersal throughout Eurasia. Today, we know that there was not a single colonization event and that the process was progressive while coping with the MIS3 abrupt climatic oscillations. Modern humans expanded into the continent by adapting to different topographic situations and by exploiting resources in diverse ecological niches. The northern part of Italy is one of the first European regions where early modern humans are documented. Here, we present the subsistence regimen adopted by the Protoaurignacian groups in two different levels in Fumane Cave based on archaeozoological data. New radiocarbon dates confirm an overlap between Uluzzian and Protoaurignacian occupations, around 42 and 41,000 cal BP, and reveal that modern humans occupied the cave from GI10 to GS9, the last level coinciding with the Heinrich Event 4. The data indicate seasonal site occupations during late spring/summer and that prey exploitation was focused mostly on ibex and chamois, killed in nearby areas. The whole faunal assemblage suggests the presence of early modern humans in a cold environment with mostly open landscapes and patchy woodlands. The estimation of net primary productivity (NPP) in Fumane, compared with other contemporaneous Italian sites, reflects how the NPP fluctuations in the Prealpine area, where Fumane is located, affected the biotic resources in contrast to known Mediterranean sites. From a pan-European perspective, the spatiotemporal fluctuation of the NPP versus the subsistence strategies adopted by Protoaurignacian groups in the continent supports rapid *Homo sapiens* dispersal and resilience in a mosaic of environments that were affected by significant climate changes.

## Introduction

Recent studies about the dispersal of Anatomically Modern Humans (AMH) across Western Eurasia reformulate prevailing ideas, especially those based on their chronological expansion and arrival in different continental areas and how colonization was influenced or motivated by the climate and broader environmental conditions that AMH had to face^1–3^. Current evidence of human remains found in Grotte Mandrin in France may attest to a much earlier presence of *H. sapiens* in SW Europe between 56.8 and 51.7 ka cal BP^4^, followed by Bacho Kiro in Bulgaria^5,6^, Grotta del Cavallo, Riparo Bombrini and Grotta di Fumane in Italy^7,8^ between ~ 45 to 41 ka cal BP. This combined cultural and biological process ultimately resulted in the gradual replacement of the late Neanderthal populations of the entire continent^8–18^. During the earliest Upper Palaeolithic, the AMH populations, coming from the Levant, progressively settled in the various areas of the continent following both the Mediterranean and Centro-European routes^13^. This process is characterized by the appearance of new cultural traits, such as symbolic material including artworks and personal ornaments^19–24^, bone and antler technology^25–29^, found across a variety of cultural technocomplexes such as the Uluzzian, Neronian, Bachokirian, Bohunician, LJR, Early Ahmarian/Kozarnikian/Protoaurignacian, etc.^30–33^. The Aurignacian is one of these first technocomplexes attributed to the spread of AMH from east to west^13,34–39^, dated to around 43–42 ka cal BP^1,12,20,40–47^. Within the Aurignacian, the earliest phase is known as the Protoaurignacian, most frequent in the Mediterranean region, while the Early Aurignacian is spread across the continental areas^13,38,39,48,49^. Differences in chronology, technology and human adaptative systems between the Proto- and Early Aurignacian, played out against the background of deterioration of the environment at the onset of Heinrich Event 4 (HE4), are still a matter of debate^1,46,49–54^. While Banks et al.^50^ proposed that the EA started at the onset of HE4, immediately after the PA (contra Barshay-Szmidt et al.^46^ and Higham et al.^53^), other studies have pointed out that some PA assemblages are dominated by cold steppe animal species and, for this reason, this technocomplex was still present during the HE4 in some regions, such as central and southwestern France^55^.

However, while in the last decade intensive work has been done to disentangle the main technological features of the Aurignacian dispersal and its complex synchronic and diachronic variability, few studies exist about the subsistence and the ecological settings and biodiversity exploited by the makers of the new technology. Research has mainly focused on the subsistence differences between Neanderthals and modern humans. On a broader scale, scholars agree that there was no significant change in the range of hunted taxa or differences in the abundance of the species by modern humans, remarking that economic diversifications can be ascribed to environmental changes that led to fundamental socio-economic developments^56^, rather than divergence in hunting strategies or dietary shifts^15,55,57–67^. However, documenting the subsistence strategies employed by early modern humans in their continental dispersal is key to understanding their success. Not only detailed information on the archaeozoology of faunal assemblages, but also high-resolution chronological, subsistence and Net Primary Productivity data are thus needed to provide accurate insights on human-environmental interactions during the early phases of modern humans on a regional scale.

**Figure 1:**
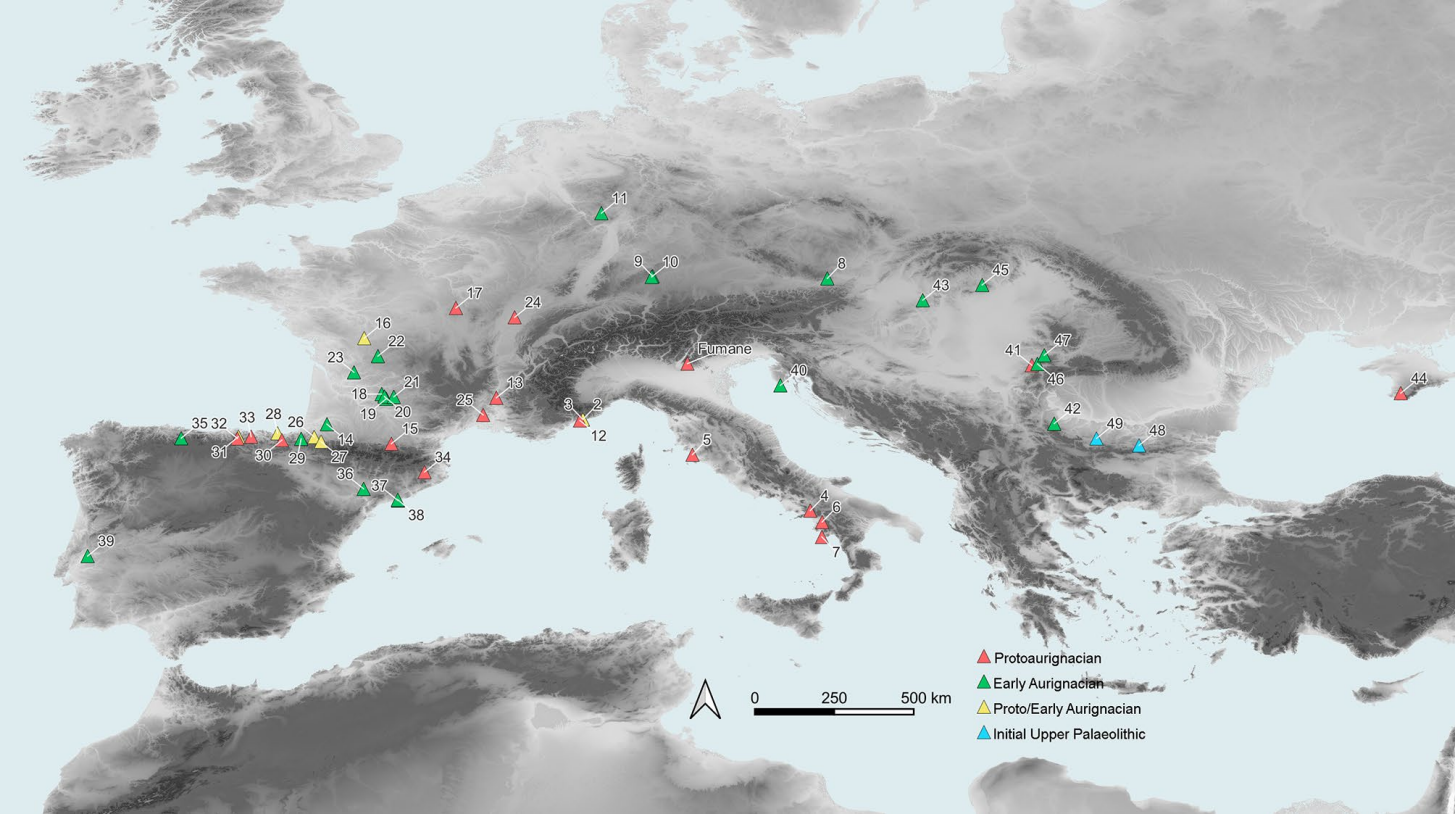
Fumane cave and Protoaurignacian, Early Aurignacian and Initial Upper Palaeolithic sites in Europe: (2) Bombrini; (3) Mochi; (4) Serino; (5) La Fabbrica; (6) Castelcivita; (7) La Cala; (8) Willendorf II; (9) Geißenklösterle; (10) Hohle Fels; (11) Wildscheuer; (12) Observatoire; (13) Mandrin; (14) Brassempouy—Hyènes; (15) Tuto-de-Camalhot; (16) Les Cottés; (17) Grotte du Renne; (18) Castanet; (19) Roc de Combe; (20) Caminade-Est; (21) Le Flageolet I; (22) Combe Saunière; (23) La Quina “aval”; (24) Trou de la Mère Clochette; (25) Esquicho-Grapaou; (26) Isturitz; (27) Gatzarria; (28) Labeko Koba; (29) Aitzbitarte III; (30) Ekain; (31) Covalejos; (32) El Castillo; (33) El Cuco; (34) Arbreda; (35) La Viña; (36) Cova Gran; (37) Canyars; (38) Cova Foradada; (39) Lapa do Picareiro; (40) Šandalja II; (41) Tabula Traiana; (42) Kozarnika; (43) Peskő; (44) Siuren I; (45) Istállóskő; (46) Tincova; (47) Româneşti; (48) Bacho Kiro; (49) Temnata. Full details of the sites are provided in the Tables S3 and S4. Map was created with QGIS 3.24 and raster files were obtained from Jarvis et al.^69^.

Fumane Cave, in north-eastern Italy, contains a long, amply dated and well-established stratigraphic sequence that includes Mousterian, Uluzzian and Protoaurignacian levels, thus making it a key site for deciphering the Middle to the Upper Palaeolithic transition in south-central Europe^68^ (Fig. 1, Fig. S1). The discovery of modern human remains associated with the A2 Protoaurignacian level^8^ and the evidence of repeated, intensive occupations by modern humans^21^, offers a unique opportunity to explore the subsistence strategies and dietary practices of modern humans in Italy. For the analytical purposes outlined above, this work focuses firstly on presenting the archaeozoological study of the faunal assemblage from the two Aurignacian levels, the Protoaurignacian in A2–A1 and the late Protoaurignacian in D3; and secondly, on presenting new radiocarbon dates for those Protoaurignacian levels and the underlying Uluzzian level (A3) to provide, for the first time, a detailed understanding of the subsistence and ecosystems exploited by modern humans in this region. Thirdly, the calculation of Net Primary Productivity (NPP) is undertaken to compare how this is correlated with the ungulate species exploited at Fumane throughout time and, also, to compare it with the NPP of other Italian Protoaurignacian sites. Finally, the archaeozoological and ecological data from other European early Upper Palaeolithic macrofaunal assemblages have been correlated with the NPP in each site to address the spatio-temporal ecological niches and prey consumed from the Black Sea to Atlantic Portugal.

## Materials and methods

### Fumane Cave

Fumane Cave is located at the foot of the Venetian Pre-Alps in the western Lessini Mountains. Excavations since 1988 in the main cave and two associated tunnels have revealed a finely-layered sedimentary succession spanning the late Middle Palaeolithic and the Early Upper Palaeolithic (macro-units A and D—Figs. S1, S2), with several features, hearths and a wide presence of lithic and faunal remains. Mousterian artifacts are associated with levels A11, A10, A9, A6, A5 and A4^68,70,71^; Uluzzian with A3^72–74^; Protoaurignacian with A2–A1, late Protoaurignacian with D6 and D3^49,75,76^, and D1d with early Gravettian^77^. In the Upper Palaeolithic sequence, numerous lithic and bone artefacts, dwelling structures with hearths and a toss zone were identified (Fig. S2a).

Currently available radiocarbon dates for the Upper Palaeolithic levels were obtained on ultra-filtered animal bone collagen and ABOx-treated charcoal samples^78,79^. Those results indicated that the earliest Protoaurignacian unit A2 dates to 41.2 and 40.5 ka cal BP, just before deposition of the Campanian Ignimbrite (not detected in the cave), while the Uluzzian seems to have ended shortly after 42 ka cal BP^72^. In this study, two new radiocarbon dates from A1 and D3 and two from A3, using ultrafiltration protocols on human-modified animal bone, were undertaken to reinforce the chronology of the early Upper Palaeolithic levels located at the entrance of the present-day cave.

Although Protoaurignacian levels A2 and A1 were excavated as single units, here they are grouped because A1 is a thin anthropogenic level in the cave entrance with horizontal bedding that is indistinguishable from A2 in innermost excavated zone. A2 thus extends throughout the whole present-day cave. The larger faunal remains were recovered and plotted individually and specimens smaller than 3 cm were recovered in general bags from each 33 × 33 cm sub-square after wet-sieving the sediments with 2 mm mesh.

### Archaeozoological analysis

The archaeozoological and taphonomic analyses of units A2–A1 (Protoaurignacian) and D3 (late Protoaurignacian) are presented here. The macrofaunal material was recovered from a 16 sq m area documented in the cave entrance and near the combustion features found during the excavations between 1991 to 2006 (Fig. S2). We have restricted our sample to all materials recovered in the front part of the cave (Fig. S2), where the stratigraphy is fine-grained, and the youngest phase is divided into several units. Also, A2 and A1 are easily distinguishable in this area. In addition to a preliminary palaeontological study of the Protoaurignacian fauna remains recovered between 1988 and 1991 was conducted by Cassoli and Tagliacozzo^80^, specimens related to some anthropogenic structures were analysed by Broglio et al.^81^. Preliminary analyses of cementochronology on ungulates were performed by Facciolo et al.^82^, and taxonomical determinations of bone retouchers were carried out by Jéquier et al.^83^. Here we provide, a complete archaeozoological and taphonomical assessment for the entire sequence of these levels, considering previous analyses and providing new data about the subsistence patterns and the ecosystems exploited by the modern human groups.

The studied material was recorded using a database purposely designed for the Subsilience project. Taxonomy was determined using the osteological comparative collection based at the EvoAdapta Laboratory of the University of Cantabria. Due to the high fragmentation and the lack of any distinctive landmark, taxonomically unidentifiable specimens were grouped according to their body size into five mammals categories: size 1: smaller than rabbit; size 2, small mammals weighing < 20 kg (lagomorphs, rodents and small carnivores); size 3, medium mammals between 20 and 100 kg (*Capra ibex*, *Rupicapra rupicapra*, *Capreolus capreolus*, *Canis lupus*); size 4, medium/large mammals between 100 and 300 kg (*Cervus elaphus*, Ursidae); size 5, large mammals of > 300 kg (*Megaloceros giganteus*, large bovines). When species determination was undistinguishable, elements were reported to their family (e.g. Cervidae, Caprinae) or genus (e.g. *Capra/Cervus*, *Rupicapra/Capreolus*, *Bos/Bison* sp.) level.

The assemblage was quantified by applying the following indices: Number of Remains (NR), Number of Identified Specimens (NISP), Minimum Number of Individuals (MNI), Minimum Number of Skeletal Elements (MNE) and Minimum Animal Units (MAU) following Marín-Arroyo^84^. For the quantification of the long bones, the coding method established by Romandini^85^ after Marean et al.^86^ was followed. Thus, each element was divided into 20 portions with a unique anatomical code indicating the lateral, medial, proximal and distal parts of the bone (Fig. S3). The repetition of codes and sides per species (considering the presence/absence of landmarks and ages) yielded the MNE. A triangular plot showing the age categories grouped into juveniles, prime adults and senile individuals was done following the mortality profiles, as proposed by Stiner^87^. Only those species represented by more than four individuals were included in the plot. Biomass calculation was made by multiplying values of useable meat (following the methodology applied in Marín-Arroyo and Gónzalez-Morales)^88^ by the MNI. The assemblage diversity was examined through the Inverse of Simpson’s Index (1/D)^89^. The higher the value, the wider the diet breadth^90^. For regional and European comparison of hunting preferences, the Inverse of Simpson’s Index with NISP and MNI was estimated, whenever possible. The ratio of high to low prey ranks to evaluate the selection of the prey hunted was also calculated. Among the determined species at Fumane, roe deer and chamois were considered low‐ranked species, while the rest of the ungulate preys were considered to be high‐ranked (giant deer, red deer, ibex and bovines [bison and/or aurochs]), in accordance with previous studies by Marín-Arroyo^91^, here adapted to the specific case of Fumane.

The ungulate mortality pattern was assessed by both dental eruption and wear stage and bone fusion. For cervids, the studies by Azorit^92^, Mariezkurrena^93^ and Tomé and Vigne^94^ were followed. For caprids, Couturier^95^, Habermehl^96^ and Pflieger^97^ were used. Once the age of death was determined, individuals were grouped into five age groups: F (Foetal/Neonatal); J (Juvenile—light worn on deciduous teeth and erupted M1); SAd (sub-Adult—moderate worn on deciduous teeth and erupted M2); Ad (Adult—all permanent teeth erupted with any or moderate wear); S (old Adult or Senile—advanced wear). The ratio between juvenile and adult individuals was estimated to measure the pressure on low-return younger prey and the exploitation prey type.

To evaluate the carcass exploitation and transport type, bivariate correlations between %MAU and different indices such as MGUI (Modified General Utility Index—Binford^98^), FUI (Food Utility Index—Metcalfe and Jones^99^), CFUI (Corrected Food Utility Index—Morin and Ready^100^) were undertaken. %MAU and Maximum Bone Density^101^ was applied to assess the influence of attrition in the faunal assemblage. To determine if the bone breakage patterns were the result of marrow extraction, correlations between %MAU and the Marrow Index^98^ were calculated. In addition, %MAU of high survival skeletal elements was correlated with the unsaturated marrow index (UMI) proposed by Morin^102^. Spearman’s correlation coefficient was used to test the quantitative validity of these correlations and determine the level of statistical significance (p) thereof. Furthermore, to overcome the limitations of previous indices on evaluating the skeletal profiles and attrition at the site, a Bayesian method based on a Monte Carlo Markov Chain sampling that uses the available skeletal information to constrain the possible degrees of attrition and carcass processing strategies was applied^103^. This method considers two parameters, alpha (α) and beta (β), that are active during the assemblage formation and history, α informs about the transport preference based on butchering efficiency in skeletal elements and it can take any value between − 1 (> axial contribution) and 1 (> appendicular contribution). The degree of attrition (β), follows the definition established by Rogers^104,105^, which related the survivorship of bone elements to their maximum bone density. This method aims specifically to overcome the problem of equifinality in skeletal profile interpretations, analysing two factors simultaneously^106^.

Every anatomical element (over 3 cm long) was examined under a LEICA S8 APO stereoscope with 10× eyepieces in search of bone surface biostratinomic and diagenetic alterations, such as cut marks (grouped as skinning, dismembering and defleshing types following Binford^107^, Galán and Domínguez-Rodrigo^108^ and Nilssen^109^), hammerstone percussion marks (including conchoidal notches^110–112^), type and angle of fracturing (fresh‐green versus old‐dry following Villa and Mahieu^113^, percussion marks including cortical percussion notches (negative flake scars); impact flakes (positive flake scars); adhering flakes, percussion pits and crushing marks^110,111,114–118^. According to diagnostic characteristics, impact flakes were defined and distinguished from those made by carnivores^119^: ventral face with the point of detachments and bulb; greater breadth than length; absence or reduction of the cortical surface. Thermoalterations colour degrees were recorded following Stiner et al.^120^. The study of bone retouchers was carried out following Mallye^121^. Carnivore activity was classified in pits, scores, punctures, furrowing, gnawing, crenulated edge and corrosion of gastric acid according to Blumenschine^122^, Domínguez-Rodrigo and Piqueras^123^, Domínguez-Rodrigo and Barba^124^. Other biological and physicochemical alterations, such as weathering^125^, root etching, insect/fungus activity, carbonate deposits, polishing^126–128^, or formation of mineral coatings (mainly mineral manganese, see Marín-Arroyo et al.^129,130^ were also recorded. Trampling marks were distinguished from butchering marks using guidelines by Blasco et al.^131^ and Domínguez- Rodrigo et al.^132^. For discerning bone surface modifications, a Leica DVM6 3D digital microscope was used to accurately distinguish the various kinds of alterations. Finally, data analysis was undertaken with R, v.4.1.0, using RStudio v. 1.2.1.

### Catchment areas study

To investigate the relationship between the ungulates exploited at Fumane during the Protoaurignacian and the surrounding environment, the catchment areas associated with the site were calculated, characterising the local relief, following the methodology described by Marín-Arroyo^133^. It must be considered that the Fumane and the adjoining valleys underwent limited morphological modifications since the mid-Upper Pleistocene. As part of the Monti Lessini fan-shaped plateau, this area is radially dissected by several valleys developed along the tectonic lines^134^ and gently dips to the South towards the alluvial plain of the Adige River. Some sections take the form of a canyon, locally called vai. To the North, summits reach 1500–1600 m. a.s.l., while the plateau ends to the West at the long and deep canyon of the Adige River. The immediate surroundings of Fumane cave have featured an ensemble of morpho-tectonic terraces connected to the bottom of the stream valley by steep slopes and rock walls. Generally, the plateau top, the summits of the ridges and the terraces were significantly affected by weathering, karstic and slope processes which led to the development of Terra Rossa-type palaeosols. During the late Last Glacial Maximum, the area was partly covered by the Adige glacier attested on the upper part of the Monti Lessini. Further, periglacial conditions activated strong erosion of palaeosols, accumulations of slope deposits, and all over the relief was interested in loess sedimentation^135^. Although the deepening of gorges and valleys continued throughout the Late Pleistocene and Holocene, there is no evidence of a deep modification of the sections of these main cuts^135,136^. Anthropogenic erosion during the Holocene strongly affected the main slopes especially starting from the Neolithic, resulting in gravelly, sandy and pelitic floods several meters thick, deposited at the bottom of the valleys and of the steepest slopes. The Western Lessini foreland includes the outlet and the lower reach of the Adige valley and the apex of the Adige alluvial megafan. This latter aggraded during the Last Glacial Maximum^137,138^.

A digital model of the present-day morphological conditions of the terrain around the site was produced. Travelling times across the territory were estimated with empirical formulae depending on distance, slope angles, movement direction (uphill or downhill) and the existence of insurmountable barriers. Besides, to define the preferred biotopes for plain and mountain species within the boundaries determined by the catchment area, a threshold value of 30% slope was fixed to differentiate areas related to one or the other group of faunal taxa. Beyond that value, grazing suitability is depleted^139^.

### Net primary productivity

Net primary productivity (NPP) is the biomass per unit of land surface and time of all autotroph organisms. Therefore, NPP represents the base of the food chain for all terrestrial ecosystems^140^. In the current study, NPP was estimated over an area of 50 km^2^ around the Fumane cave and in the surrounding area of 26 European archaeological sites with dated Protoaurignacian, Early Aurignacian and early Upper Palaeolithic techno-complexes associated with faunal remains. NPP was estimated with the Lund-Postdam-Jena General Ecosystem Simulator (LPJ-GUESS) v.4.0. LPJ-GUESS is a coupled biogeography-biogeochemistry model which incorporates process-based representations of terrestrial vegetation dynamics at regional and global scales^133,141^. Vegetation composition in the model results from growth and competition for light, space and soil resources among plant communities according to specific photosynthetic rates, stomatal conductance, phenology, allometric calculations, and carbon and nutrient allocation according to specific Plant Functional Types (PFTs)^142^. PFTs are a group of species with similar bioclimatic limits, morphology, phenology, photosynthetic pathway and life history strategy^142,143^. Different PFTs can be incorporated and simulated in LPJ-GUESS according to their specific functional traits; in the current study, we used the standard global PFT set described in Smith et al.^142^ with the ‘cohort’ mode. The model was run in 100 replicate patches with an area of 0.1 ha, and we did not use nitrogen limitation because nitrogen deposition is unknown for the Pleistocene^144^. For details on the global parameters used, see Table S1.

The input climate variables for LPJ-GUESS are monthly temperature (°C), precipitation (mm/month), incoming shortwave radiation (Wm^−2^), and rainy days (days/month). These input data were obtained from the HadCM3B-M2.1 general circulation model^145^ after performing a delta bias correction with the CRU v.4 datasets^146^. We used the HadCM3B-M2.1 general circulation model because previous studies compared this palaeoclimatic model with the palynological record obtained from different European regions, providing evidence for the accuracy and reliability of these climatic simulations in the Northern Hemisphere^10,147,148^. The model also used, as input data, the atmospheric carbon dioxide concentration (ppm) obtained from Lüthi et al.^149^ and the specific soil classes obtained from Zobler^150^. All simulations were initialised with ‘bare ground’ conditions, and the model was spun up for 500 years until the simulated vegetation was in approximate equilibrium. This spin-up phase used palaeoclimatic conditions between 55 and 54.5 ka BP^145^. After that, the model was run at a monthly resolution spanning between 54.5 and 30 ka BP. The NPP of the Protoaurignacian units of Fumane were compared with the other archaeological Mousterian and Uluzzian units of Fumane with a Mann–Whitney–Wilcoxon test. To assess whether fluctuations in the estimated NPP affected the species diversity recovered in each archaeological unit of Fumane, a Spearman’s rank correlation test was used.

## Results

### Chronology

**Figure 2:**
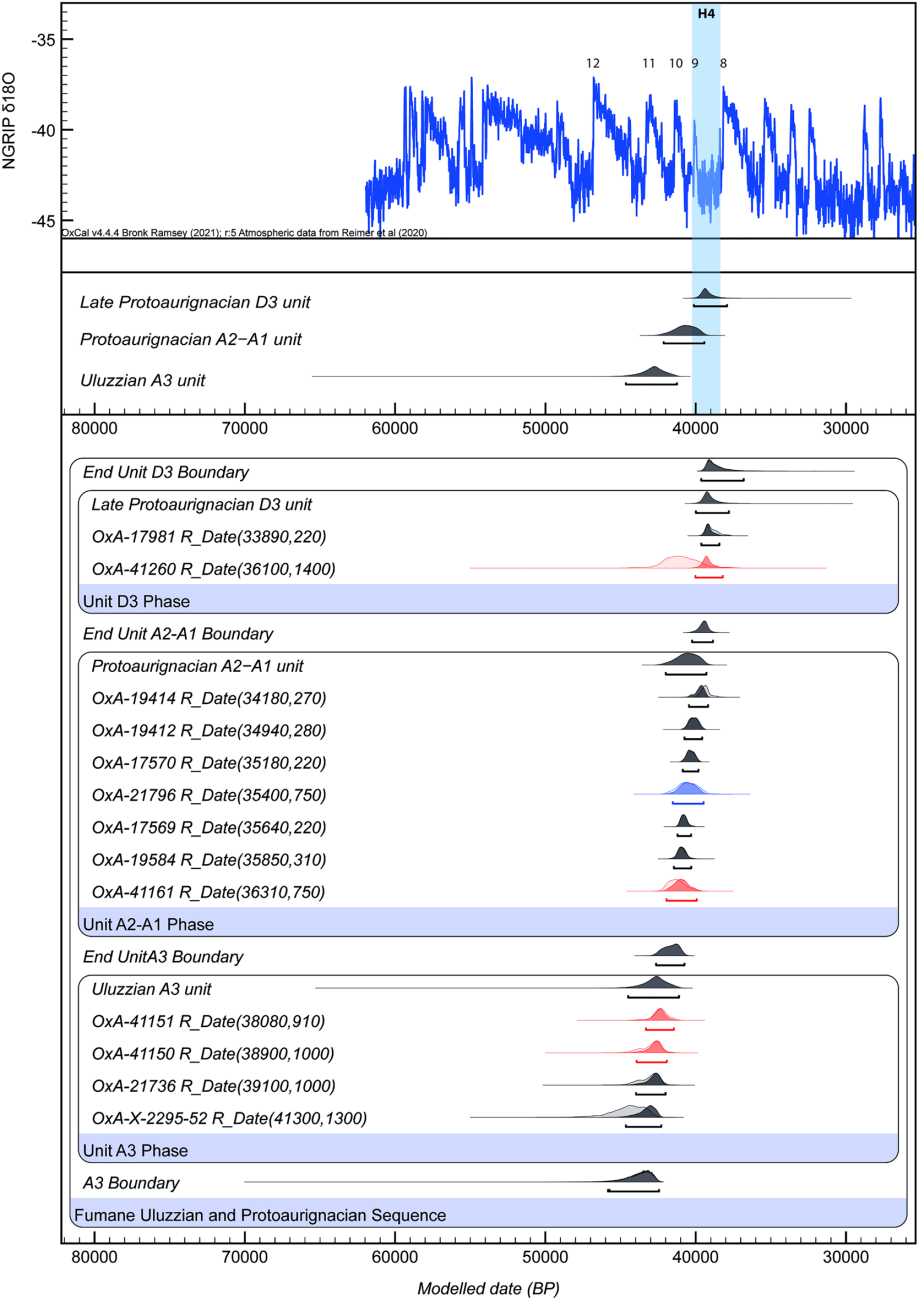
Chronological model of the Uluzzian, Protoaurignacian and late Protoaurignacian units of Fumane calibrated against IntCal20^152^ in Oxcal v.4.4^151^. All available radiocarbon dates of these units, including the new dates obtained in this work, are included in the model. In grey: dates obtained on charcoals with the ABOx pre-treatment^78,79^; in blue: dates obtained on bone collagen with the ultrafiltration pre-treatment^78^; in red: the dates obtained in this study.

The results of the new dates are provided in Table 1. Figure 2 shows the Bayesian age model built using OxCal4.4.2 software^151^ with the INTCAL20 calibration curve^152^. Considering the stratigraphic information of Fumane, the dates were modelled in a Sequence model with stratigraphic units represented as Phases from A3 (Uluzzian) and A2–A1 and D3 units and, with start and end Boundaries. The difference between the probability density functions of the start and end boundaries was also calculated to estimate the likely duration of the phase. All radiocarbon determinations were given a 5% prior likelihood of being an outlier within the General t-type Outlier Model^151^, so that the model could test their reliability. Convergence was greater than 95% and the model agreement index was 81 (Table 1, Code S1). Only date, OxA-41260, with a higher standard deviation, showed a lower convergence (37%). The reason for this larger uncertainty is due to the lower collagen yield that implied larger processing backgrounds when measuring the 14C content, this being lower when dated by AMS. The Bayesian results were compared with the Greenland ice-core oxygen isotope record (NGRIP)^153–155^ record to correlate each cultural phase with the stadial and interstadial climatic phases. The Bayesian model was run five times and the results were compared to check the consistency. They disclosed acceptable reproducibility levels when compared.

**Table 1:** Radiocarbon dates of the Protoaurignacian and Uluzzian units of Fumane Cave obtained in this study.

ID	Unit	Square	Species	Element	Taphonomic modification	Lab-ID	14C Age (BP)	%Yield	%C	δ^13^C	δ^15^N	C:N
GT703	D3d	78g	*Capra ibex*	Humerus	impact notch, cut-marks	OxA-41260	36,100 ± 1400	0.4	50	− 18.9	6	3.3
GT1088	A1	66d	*Capra ibex*	Tibia	cut-marks	OxA-41161	36,310 ± 750	3.4	42.9	− 19.5	4.1	3.2
14	A3_SI	77f	*Bos/Bison*	Metatarsal	impact notch, cut-marks	OxA-41151	38,080 ± 910	4.1	46.2	− 19.6	7.6	3.2
R523	A3	66h	*Cervus elaphus*	Phalanx I	chop mark	OxA-41150	38,900 ± 1000	6.3	45.7	− 20.3	4.4	3.3

The results of the Bayesian model indicate the Uluzzian occupation dates to 44,500 to 41,100 cal BP, the Protoaurignacian A2–A1 unit between 42,000 and 39,250 cal BP and the late Protoaurignacian D3 unit between 40,000 and 37,750 cal BP (95.4% likelihood) (Fig. 1). The end date of the Uluzzian occupation falls within the limit for this phase in the Italian Peninsula around 41,000^156^. In Fumane, the new chronology reveals an overlap between Uluzzian A3 and Protoaurignacian A2–A1 unit, around 42 and 41,000 cal BP. Likewise, a short overlap is observed between the Protoaurignacian and the late Protoaurignacian around 40 and 39,000 cal BP. This data set confirms the previous chronology^79^, with the Uluzzian coinciding with GI11, and the Protoaurignacian covering GI10 to the end of GI9. The late Protoaurignacian D3 unit lasts during the GS9, corresponding with the Heinrich Event 4.

### Faunal assemblage and origin of the deposit

In total, the two units yielded 12,907 remains. 4.6% of the elements were identifiable to taxa, 4.9% identified only to mammal body size, and 90.6% were non-identifiable specimens. From the whole assemblage, 8.4% of the items were anatomically identified (Table 2). Due to the state of fragmentation of the assemblage (Fig. S4), only a minimum number of 226 elements (MNE) were quantified and a total of 53 MNI, including 14 different ungulates (n = 6), carnivores (n = 5), rodents (n = 1), leporids (n = 1) and an undetermined bird taxon. The data of NISP, MNE and MNI values per level and species are presented in Table 2.

**Table 2:** Number of identified specimens (NISP), minimum number of elements (MNE), minimum number of individuals (MNI) and biomass of the Protoaurignacian units.

Unit A2–A1	NISP	%NISP	MNE	MNI						%MNI	Biomass (in kg)
Taxon				F	J	SAd	Ad	S	Total		
*Bos/Bison* sp.	3	0.7	3		1		1		2	8	666.8
*Capra ibex*	136	30.2	63	1	2	1	4	1	9	38	329.68
*Rupicapra rupicapra*	54	12	28		2		2		4	17	143.36
Capridae (size 3–4)	52	11.6									
*Megaloceros giganteus*	8	1.8	7		1		1		2	8	146.69
*Cervus elaphus*	44	9.8	27		2		3		5	21	325.05
*Capreolus capreolus*	18	4	8		1		1		2	8	20
Cervidae (size 4–5)	31	6.9									
*Capra/Cervus*	12	2.7									
*Rupicapra/Capreolus*	50	11.1									
Total Ungulata	408	90.7	136	1	9	1	12	1	24		
*Lynx lynx*	1	0.2	1				1		1		
*Ursus* sp.	1	0.2	1				1		1		
*Canis lupus*	8	1.8	4		1		1		2		
*Vulpes vulpes*	5	1.1	3				1		1		
Total Carnivora	15	3.3	9		1		4		5		
*Lepus* sp.	1	0.2	1				1		1		
*Castor fiber*	2	0.4	1				1		1		
Total Lagomorpha and Rodentia	3	0.7	2				2		2		
Total *Aves* sp.	24	5.3	5				1		1		
Sub total	450	100	152	1	10	1	19	1	32		
Ungulate indeterminate	150										
Carnivore indeterminate	13										
Mammal size 1	0										
Mammal size 2	17										
Mammal size 3	185										
Mammal size 4	73										
Mammal size 5	39										
Non-identifiable	10,159										
Grand total	11,086										

Unit D3	NISP	%NISP	MNE	MNI						%MNI	Biomass (in kg)
Taxon				F	J	SAd	Ad	S	Total		
*Bos/Bison* sp.	1	0.7	1				1		1	7	400
*Capra ibex*	27	19.4	22		2		2		4	27	143.36
*Rupicapra rupicapra*	30	21.6	21		2	1	2		5	33	186.36
Capridae (size 3–4)	20	14.4									
*Megaloceros giganteus*	4	2.9	4				1		1	7	88
*Cervus elaphus*	10	7.2	4				1		1	7	75
*Capreolus capreolus*	7	5	7		2		1		3	20	28
Cervidae (size 4–5)	4	2.9									
*Capra/Cervus*	5	3.6									
*Rupicapra/Capreolus*	12	8.6									
Total Ungulata	120	86.3	59		6	1	8		15	15	
*Canis lupus*	7	5	7		1		1		2		
*Vulpes vulpes*	7	5	5		1		1		2		
*Martes foina*	1	0.7	1				1		1		
Total Carnivora	15	10.8	13		2		3		5		
Total *Aves* sp.	4	2.9	2				1		1		
Sub total	139	100	74		8	1	12		21		
Ungulate indeterminate	31										
Carnivore indeterminate	3										
Mammal size 1	1										
Mammal size 2	15										
Mammal size 3	73										
Mammal size 4	22										
Mammal size 5	7										
Non-identifiable	1530										
Grand total	1821										

*F* Foetal/neonatal, *J* Juvenile, *SAd* sub-adult, *Ad* adult, *S* senile.

Among the herbivores, *Capra ibex* and *Rupicapra rupicapra* are the most abundant taxa, followed by *Cervus elaphus* and *Capreolus capreolus*. *Megaloceros giganteus* and large bovines (*Bos primigenius/Bison priscus*) appear occasionally in both levels. Among the carnivores, the diversity of species is represented by a few remains of each species. *Canis lupus* and *Vulpes vulpes* are the most common species, followed by an element each of *Lynx lynx, Ursus* sp*.* and *Martes foina* along the sequence. Leporids appear exclusively in A2–A1 Unit. Several bird elements are represented in both units, mostly in A2–A1.

The taphonomic study is key for discerning the origin of the deposit and the main bone accumulators. Previous studies confirmed that mammals, and especially ungulates, recovered in the Mousterian and Uluzzian units at Fumane were mainly brought to the site by humans attested by the significant amount of butchering marks, fresh bone breakage, thermoalteration and skeletal profiles representation of the herbivores consumed^157–159^. Although carnivore activities were documented, it was in a limited percentage in comparison with the anthropogenic activity.

**Figure 3:**
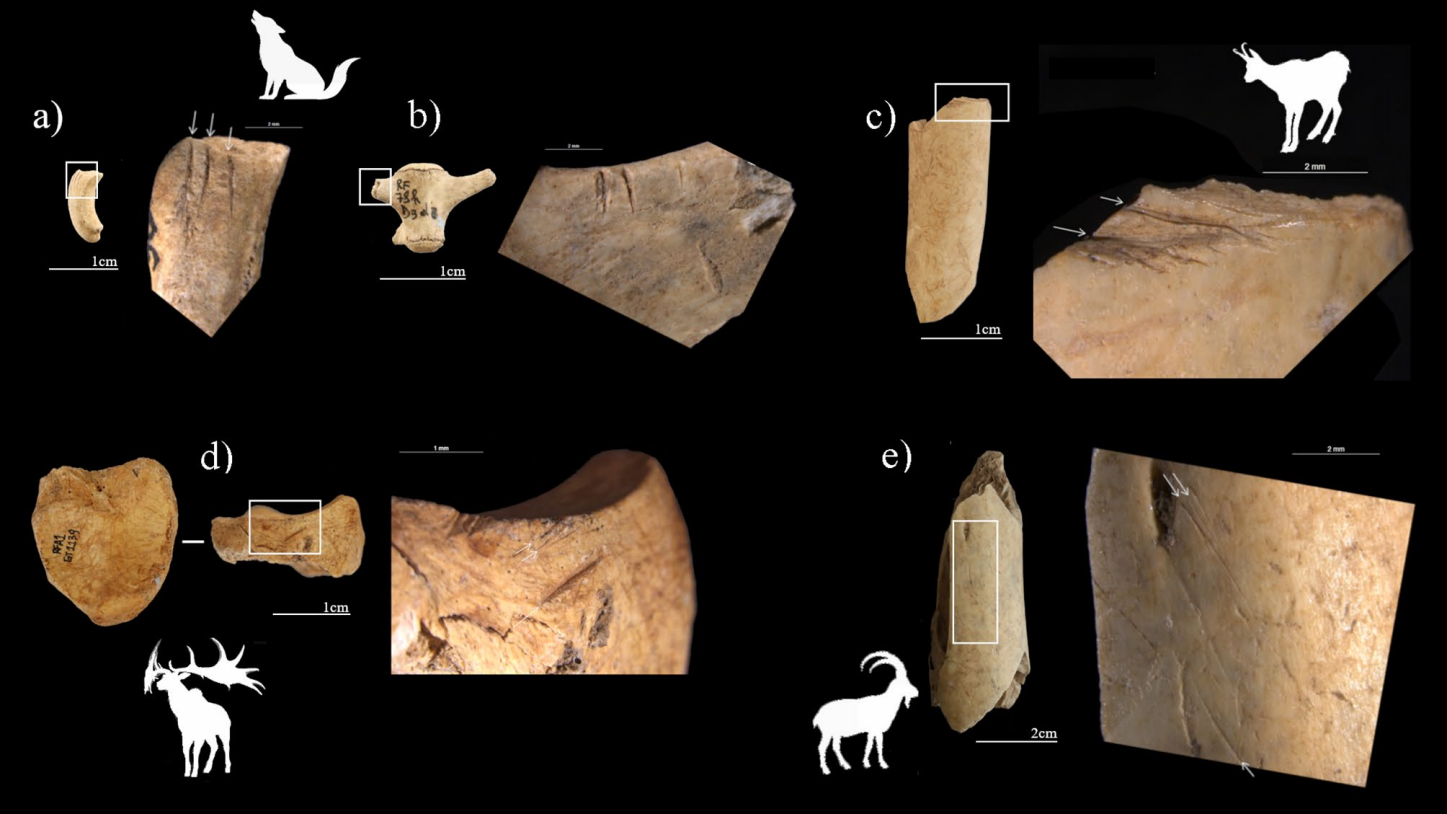
Anthropogenic modifications on mammals found in the Protoaurignacian units of Fumane: (**a**) sesamoid with skinning marks and (**b**) wolf lumbar vertebra with cut marks on the traversal processus; (**c**) chamois tibia shaft with deep and transversal cut marks; (**d**) giant deer second phalanx with skinning incisions; (**e**) ibex femur with longitudinal and oblique filleting cut marks.

In both Protoaurignacian occupations, anthropogenic modifications were identified on herbivores, including butchering activities, deliberate breakage activities (percussion marks—mainly impact flake—and percussion notches) and thermoalterations (Table 3). Fresh breakage marks are mostly found on the ibex limb bones and phalanges and red deer long bones. In addition, more percussion marks were observed on mammal size 3 and isolated bone flakes were identified among the undetermined assemblage. Regarding cut marks, A2–A1 unit reveals greater abundance in contrast with unit D3. Incisions are predominant, indicating skinning, defleshing and disarticulation activities at the site. Scrape marks related to periosteum removal are also documented. Cut marks appear on different anatomical portions, with a predominance of obliquely oriented incisions on the shafts, being scarce on the cranial and axial skeleton. In addition to ungulates, cut marks were found on carnivores, revealing fox skinning and wolf defleshing for likely fur extraction (Table 3, Fig. 3). Even in unit A2–A1, a cut mark was identified on the wing bone of a bird. Bird exploitation was already documented in the Mousterian units of Fumane revealed on species relatable and not relatable to feeding or utilitarian uses where cut marks and fractures were observed on wings, indicating the intentional removal of large feathers by Neanderthals^160^, and also on meat-bearing anatomical portions^158,161,162^.

**Table 3:** Anthropogenic activities recorded in the faunal assemblage of the Protaurignacian units.

A2–A1	NISP	PM	CM	CM + PM	%BM	R	%B
Taxon							
*Bos/Bison* sp.	3		1	1	66.7	1	–
*Capra ibex*	136	8	12	1	15.4	1	4.4
*Rupicapra rupicapra*	54	2	8		19	1	7.4
Capridae (size 3–4)	52	2	5		11.5		5.7
*Megaloceros giganteus*	8	1	1		37.5		–
*Cervus elaphus*	44	4	1		11.4	1	9
*Capreolus capreolus*	18						50
Cervidae (size 4–5)	31		1		3.2		
*Capra/Cervus*	12		1	1	16.7		
*Rupicapra/Capreolus*	50	3	7		18	1	14
*Canis lupus*	8		1		12.5		
*Vulpes vulpes*	5		1		20		20
*Aves* sp.	24		1		4.2		
Ungulate indeterminate	150	2	13	1	11		8
Carnivore indeterminate	13						15.3
Mammal size 2	17		2		11.8		17.6
Mammal size 3	185	7	24	1	16.2	1	15.1
Mammal size 4	73	4	4	1	12.3	2	4.1
Mammal size 5	39	2	2		10.3		
Non-identifiable	10,159	89	55	2	1.5		39.8
Total	11,086	124	140	8	2.5	8	37.1

D3	NISP	PM	CM	CM + PM	%BM	R	%B
Taxon							
*Capra ibex*	27	2	2	1	18.5	2	
*Rupicapra rupicapra*	30		3		10		16.6
Capridae (size 3–4)	20		2		10		
*Cervus elaphus*	10					1	
Cervidae (size 4–5)	4	2	1		75		
*Capra/Cervus*	5	1	1		40		
*Rupicapra/Capreolus*	12			2	16.7		
*Canis lupus*	7		3		42.9		
Ungulate indeterminate	31		4		12.9	1	6.4
Mammal size 2	15		1		6.7		6.6
Mammal size 3	73	3	6	1	13.7		6.8
Mammal size 4	22	1			4.5		4.5
Non-identifiable	1530	22	14		2.4		28.7
Total	1821	31	37	4	3.9	4	24.9

*PM* percussion marks, *CM* cut marks, *CM* + *PM* cut marks + percussion marks, *% BM* % butchering marks on the number of remains, *R* retouchers, *%B* % burned bones.

Among the complete assemblage, 37% and 24% in A2–A1 and D3 units, respectively showed thermoalteration and different burning categories from brown to black colouration were recognised (Table 4). In both units, categories 3 (charred bones) and 5 (calcinated bones) are the most recurrent. Although several bones with double or multiple colourations were recovered, single-coloured thermal alterations are prevalent.

**Table 4:** Number of remains (NR) classified in each burning category.

		Thermoalteration codes					
		0	1	2	3	4	5
		Unburned	Brown/black	Brown	Black	Grey	White
A2–A1	NR	6865	505	12	2825	156	1385
	%NR	58.4	4.3	0.1	24	1.3	11.8
D3	NR	1356	74	3	383	21	245
	%NR	65.2	3.5	0.1	18.4	1	11.8

Apart from butchering marks on bone surfaces, 12 bone hammers, likely used for retouching flint artefacts, were recovered. These retouchers were manufactured on ibex, chamois, red deer and large bovine long bone shafts as previously observed in other Fumane units^83^. Likewise, the other five retouchers were identified in shafts of medium and large-sized animals but the fragmentation prevented the taxa identification (Table 3).

Evaluating the role that carnivores might have had in the Protoaurignacian deposits, the bone surface modifications evidenced by these agents in both units are limited. The few carnivore marks are only identified in mammal size 3 (frequently on caprids), unidentified remains on long bone shafts and just a few on flat bones and epiphysis. Any other taxa do not bear carnivore bone modifications (Table 5). Pits, scores, furrowing and digestion were the kinds of modifications that were recorded. Although a few remains presented human and carnivore modifications, no overlap between them has been observed. This limited presence might imply the occasional scavenging of human leftovers. Therefore, the Protoaurignacian units are mostly linked to human activity, as are the previous Uluzzian and Mousterian occupations documented at Fumane.

**Table 5:** Number of remains (NR) and its percentage for the biostratinomic and diagenetic alterations recorded in the Protoaurignacian units.

			A2–A1		D3	
			NR	NR%	NR	NR%
Alterations	Biostratinomic	Carnivore marks	24	0.2	21	1.2
		Rodent marks	6	0.1	5	0.3
		Weathering	136	1.2	42	2.3
	Diagenetic	Water dissolution	25	0.2	11	0.6
		Concretions	167	1.5	42	2.3
		Root marks	507	4.6	72	4
		Manganese staining	331	3	150	8.2
		Iron mineral staining	117	1.1	6	0.3
		Total	11,086		1821	

### Palaeoeconomic behaviour of the Protoaurignancian groups

The taphonomic results of both Protoaurignacian units is mostly linked to human activities at Fumane. Consequently, the taxa represented can be interpreted as part of human subsistence. Medium/large-size mammals such as *Cervus elaphus* and medium-size such as *Capra ibex, Rupicapra rupicapra* and *Capreolus capreolus* played an essential role in the diet of the human groups at that time. In terms of MNI, during A2–A1 unit, ibex, red deer and chamois, are the most exploited species with 38%, 21% and 17%, respectively, followed by limited roles of aurochs, roe deer and giant deer, with a total of 8%, while during D3 unit occupations, red deer decreases significantly to 7% and roe deer increases up to 20%. Both montane caprine species continue playing the most significant role in the human diet, with a slight decrease in ibex (27%) and a remarkable increase in chamois (33%). Giant deer maintains its representation with 7% of the ungulates (Table 2). In terms of biomass, bovines in both units, despite their low MNI, played an important role in the protein input into the human diet because of their size. In A2–A1 unit, bovines are followed by red deer and ibex, almost in identical proportions. It is remarkable, how unit D3 reflects a change in subsistence, likely motivated by the environmental conditions during that occupation, after bovine, the core of the human diet is contributed by chamois and ibex (Table 2).

After calculating the Inverse of the Simpson index, with NISP and MNI for both units, when comparing the MNI values, the results indicate a similar diet breadth during both Protoaurignacian occupations reflecting the exploitation of caprinae and cervids. The value in unit D3 is slightly lower (4.25) than in unit A2–A1 (4.3) and both values likely reflect the exploitation of a high-relief, mosaic landscape surrounding the cave. The NISP values for D3 (3.47) is higher than for A2–A1 (2.44), which could be partially influenced by the assemblage fragmentation, or other factors such as the duration of occupation at that time, seasonality or human group size, among other factors.

### Ungulate mortality profiles and seasonality

**Figure 4:**
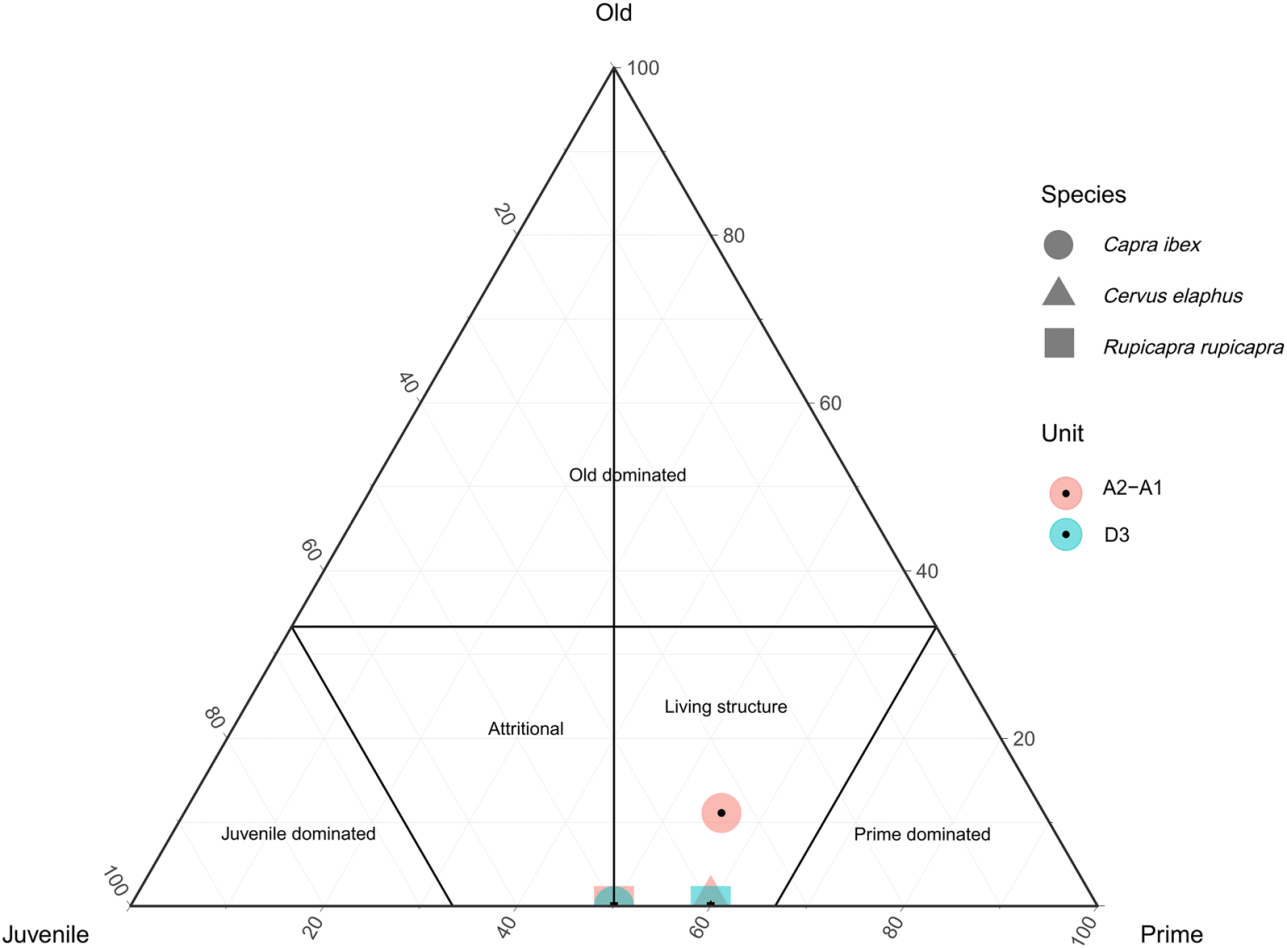
Ternary plot of main ungulate taxa—ibex, chamois and red deer for Protoaurignacian units A2–A1 and D3 assemblages.

During both units, there is a predominance of prime-age individuals (54 and 53%), followed by juveniles (46 and 47%) in A2–A1 and D3 units, respectively (Table 2). Only foetal/neonatal, subadult and senile individuals are exclusively found in A2–A1 unit (one each). Excluding those, prime-age individuals predominate over juveniles in both units. By looking at the ratio between juvenile and adult individuals, the results show similar values: 0.85 in A2–A1 unit and 0.88 in D3 unit. Based on the ternary distribution of the age profiles, for ibex and red deer a catastrophic mortality profile was observed in unit A2–A1 and between an attritional and a catastrophic one, for ibex, in unit D3. For chamois, we determined a catastrophic profile in unit D3, and between attritional and catastrophic in unit A2–A1 (Fig. 4). Regarding the seasonality data obtained from the ungulate prey, the ibex and red deer dental eruption allowed identification of late spring/early summer kills in unit A2–A1, as well as for infantile chamois individuals, which, in both units, showed a decrease in kills at the end of spring/early summer, coinciding with the annual birthing and infant suckling periods. These ungulate profiles and seasonal data would suggest an exploitation pattern of herds of females with their young calves. Results are in accordance with previous cementochronology data obtained previously^82^.

### Skeletal profile representation

**Figure 5:**
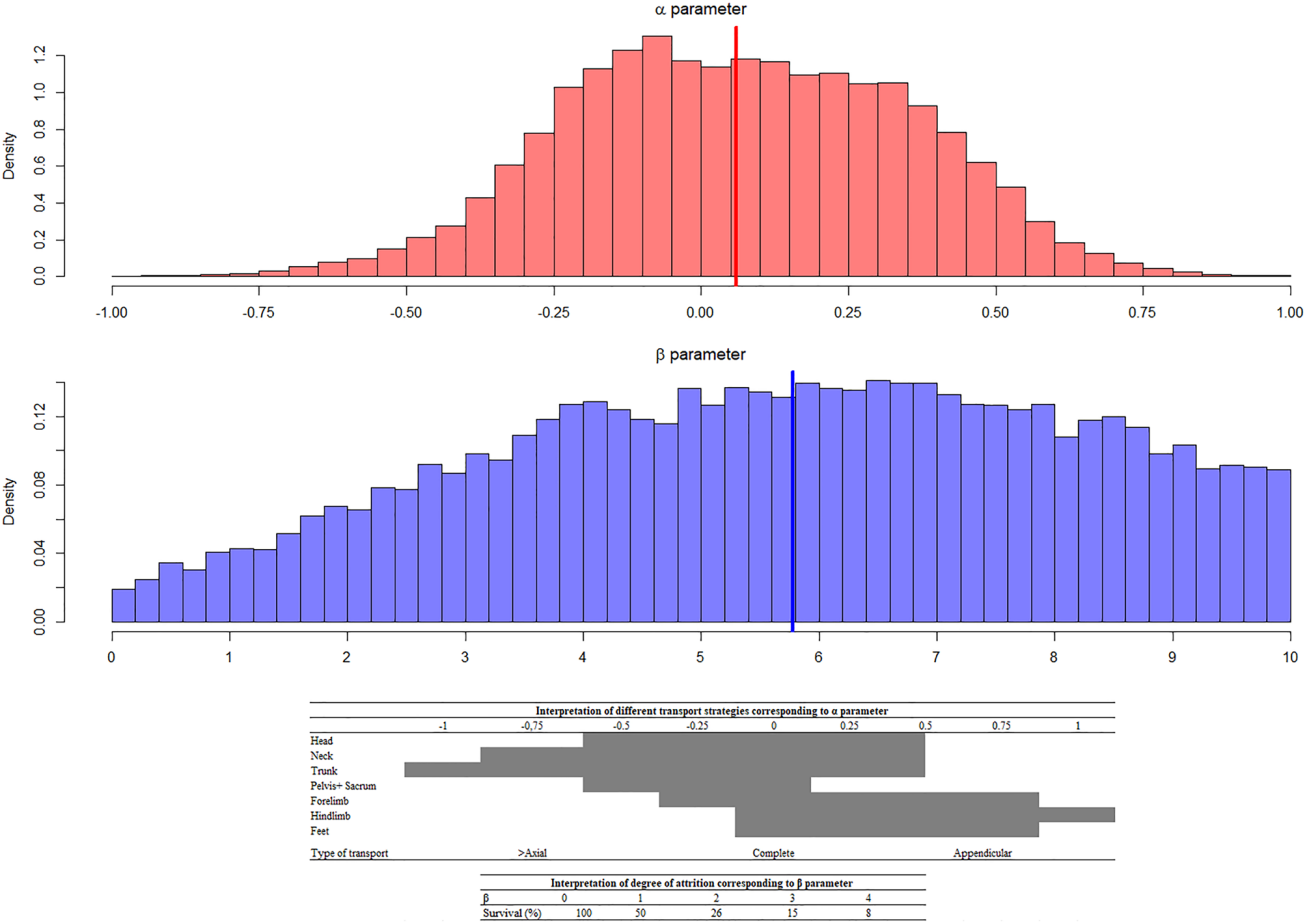
Results of the skeletal profiles for *Capra ibex* at A2–A1 unit in Fumane showing the posterior distribution function and the values of the α and β parameters. The interpretation of the parameters in the Bayesian model is explained at the bottom of the figure.

For the analysis of the skeletal profiles, a Bayesian method (BaskePro) following Marín-Arroyo and Ocio^103^ (R code in Marín-Arroyo et al.^106^) was applied to disentangle the prey’s transport and the attrition that occurred at each unit. This new method was applied only with ibex from unit A2–A1, as it was the only species with an MNE higher than 50 corresponding to nine different individuals (Fig. 5). Unfortunately, due to the low number of anatomical elements in the other ungulate prey and units, this analysis was not undertaken. For ibex, the results indicate that a complete transport of the prey was undertaken (α = 0.06), suggesting a likely nearby kill. The attrition value (β = 5.78) is extremely high, indicating that less than 8% of the original assemblage is preserved (Fig. 3).

There were abundant signs of marrow extraction for ibex at A2–A1 unit. The degree of fragmentation (measured as the quotient between NISP and MNE) correlates positively and significantly with Marrow Index^98^. Also, Spearman’s correlation coefficient between Grease Index and %MAU shows a positive and significant correlation with ibex. Bone density was also determined for this species (Table 6), which correlates with the results obtained by the BaskePro method.

**Table 6:** Results of the statistical correlations for *Capra ibex* at A2–A1 unit in Fumane cave.

*Capra Ibex*—unit A2–A1		*p*
Indices	Spearman	
Bone density	**0.79406**	**0**
Meat	− 0.06623	0.076
Marrow	**0.74829**	**0**
Grease	**0.62947**	**0.002**
FUI	0.00345	0.988
CFUI	0.10519	0.659
MGUI	0.11995	0.577
UMI	**0.79466**	**0.001**

Bivariate correlations are done between %MAU and bone density and the following indices: Meat, Marrow, Grease, FUI (Food Utility Index), CFUI (Corrected Food Utility Index), MGUI (Modified General Utility Index), UMI (Unsaturated Marrow Index). In bold significant results.

### Mobility patterns

**Figure 6:**
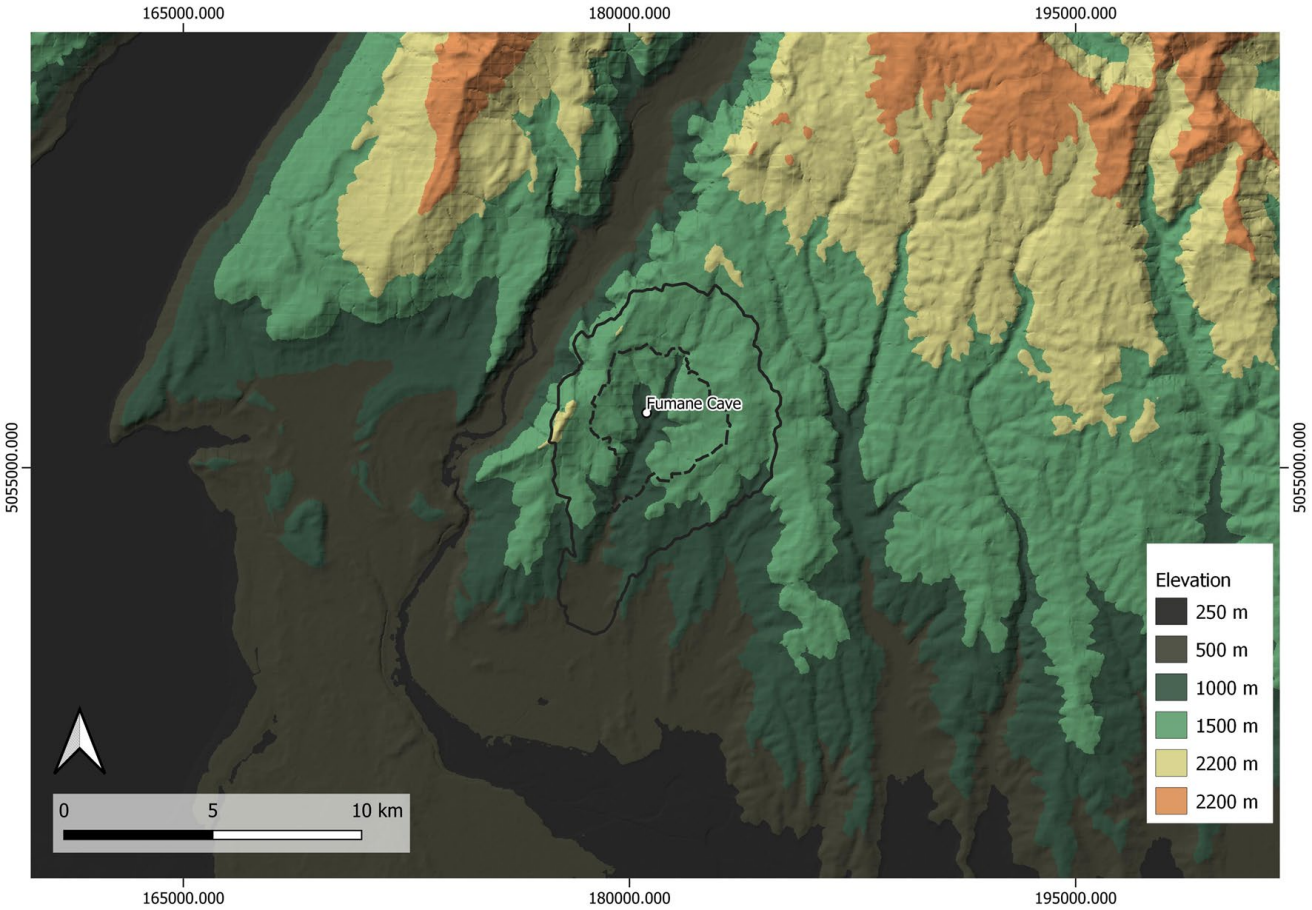
Catchment areas with Isochrones for 1.2- and 2.15-h distances originating in Fumane in a northerly direction, calculated according to the exposed methodology. Digital Terrain Model prepared from PNOA MDT05 (generated from the 1st LiDAR Coverage with a 5 m mesh pitch), sheets 28, 29, 52 and 53. ETRS89 Huso 30N projection. National Geographic Information Center (http://centrodedescargas.cnig.es/CentroDescargas/catalogo.do?Serie=LIDAR). Map done by J. García Sánchez.

Table 7 shows the percentage of areas below and above 30% slope calculated for 1.2 h and 2.15 h from the cave. Figure 6 shows the catchment areas corresponding to different travel times around Fumane.

**Table 7:** Results of the catchment areas calculated for Fumane Cave as a central point.

Unit A2–A1	NISP	MNI	%NISP	%MNI		Unit D3	NISP	MNI	%NISP	%MNI
*Bos/Bison* sp.	3	2	1.1	8.3		*Bos/Bison* sp.	1	1	1.3	6.7
*Megaloceros giganteus*	8	2	3.0	8.3		*Megaloceros giganteus*	4	1	5.1	6.7
*Cervus elaphus*	44	5	16.7	20.8		*Cervus elaphus*	10	1	12.7	6.7
*Capreolus capreolus*	18	2	6.8	8.3		*Capreolus capreolus*	7	3	8.9	20.0
*Capra ibex*	136	9	51.7	37.5		*Capra ibex*	27	4	34.2	26.7
*Rupicapra rupicapra*	54	4	20.5	16.7		*Rupicapra rupicapra*	30	5	38.0	33.3
**Mountain**	**190**	**13**	**72.2**	**54.2**		**Mountain**	**57**	**9**	**72.2**	**60.0**
**Plain**	**73**	**11**	**27.8**	**45.8**		**Plain**	**22**	**6**	**27.8**	**40.0**
Hunting preference	2.60	1.18				Hunting preference	2.59	1.50		

Altitude (m)	72 min (1.2 h)					129 min (2.15 h)				
	km^2^					km^2^				
	Plain areas	Mountainous	Total	Plain %	Mountain %	Plain areas	Mountainous	Total	Plain %	Mountain %
350	13.19	2.34	15.53	84.94	15.06	53.03	5.93	58.96	89.94	10.06

The hunting preference, obtained with the NISP and MNI of both Protoaurignacian units as they ration between mountain versus lowland habitat species. Significance values are in bold.

As can be seen in Fig. 6, Fumane is located on a mountain gorge suitable for mountain species, but also for forested areas, where the nearby massif elevation is lower than 1.500 m. This might explain the dual exploitation of mountain species, such as ibex and chamois in the surrounding of the cave during units A2–A1 (54% MNI) and D3 (60% MNI), followed by species that could have lived in woods such as red deer, giant deer and roe deer. Plains that could have harboured open humid woods—as one of the preferred habitats for bovines—are located further away and this might explain the lower representation of them in both units.

The mountainside cave location also might explain a low amount of ibex carcass processing at the kill sites, which is suggested by the mainly complete transport of these prey during the Protoaurignacian. The hunting preference, calculated as the ratio between mountain and fluvial plain habitat species, does not reveal drastic changes between units (Table 7). Only for MNI values is the ratio higher in D3 due to a greater percentage of mountain-dwelling individuals in comparison with A2–A1.

Despite the fact that within a 1.2 h walk from the site, the catchment area above a 30% slope makes up only 15%, this area would have provided access to chamois and ibex (although both taxa might have lived above that slope, depending on the season and vegetation cover), confirming the use of the nearby environment by the human groups. Within 2.15 h there is a shift of 5% of the mountain area in favour of the plain areas, but this small shift does not affect the hunting preferences undertaken by the different human groups occupying the cave (Table 7). In summary, within the distance of 1.2 h from the cave, human groups during the Protoaurignacian would have had accessibility to all types of ungulate taxa, mostly exploiting those adapted to the mountain and forested landscapes. The sources of the raw materials near the site^68^ also support the picture these locally limited human mobility patterns.

### Net primary productivity results

**Figure 7:**
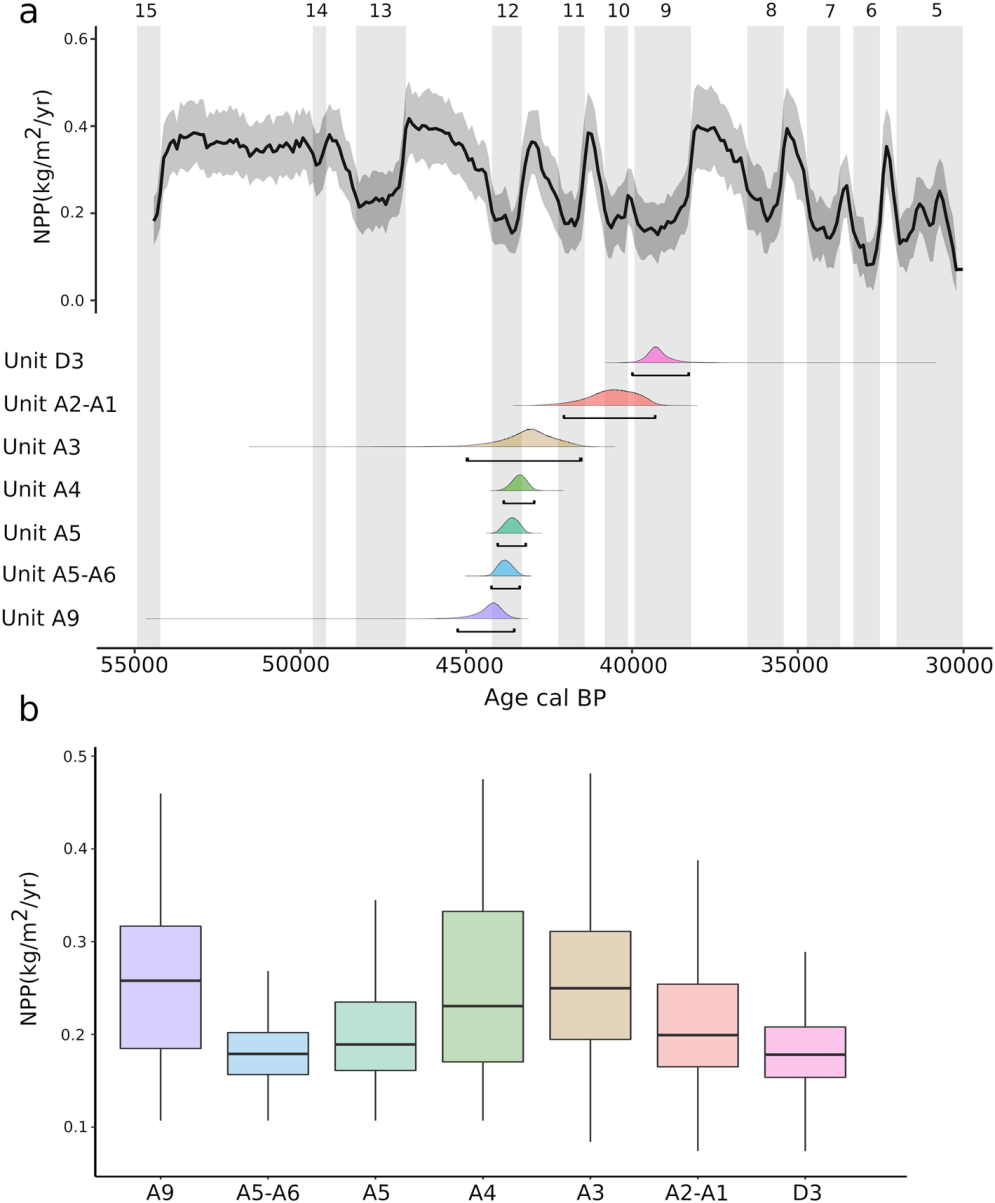
(**a**) Temporal evolution of the Net Primary Productivity (NPP) in Fumane with the Probability Distribution Function (PDF) at 95% CI of each archaeological unit. Vertical shaded bars indicate the stadial phases. (**b**) Box and whisker plot with the estimated NPP in each archaeological unit (see Tables S6 and S7, Code S2 for Fumane radiocarbon dates modelling).

Considering the different Mousterian (A11, A10, A9, A5–A6 and A4), Uluzzian (A3) and Protoaurignacian units that have revealed human occupations, the results indicate that in Fumane, during the interstadial conditions of MIS3, the Net Primary Productivity (NPP) was 0.327 kg/m^2^/year on average (SD 0.08). However, during the stadial phases, the mean NPP dropped to 0.189 kg/m^2^/year on average (SD. 0.09). During the Protoaurignacian occupation recorded in unit D3, the mean NPP was 0.181 kg/m^2^/year (SD 0.02) and, in A2–A1 unit, the NPP was 0.218 kg/m^2^/year (SD 0.06). Accordingly, the NPP of the Protoaurignacian units (A2–A1 and D3) of Fumane are significantly lower than that of the previous late Mousterian (A4) and Uluzzian (A3) units (Fig. 7). Thus, the occupation of the first modern humans in Fumane was coincident with a decreasing trend of the NPP likely associated with Heinrich 4 environmental conditions (Fig. 7).

**Figure 8:**
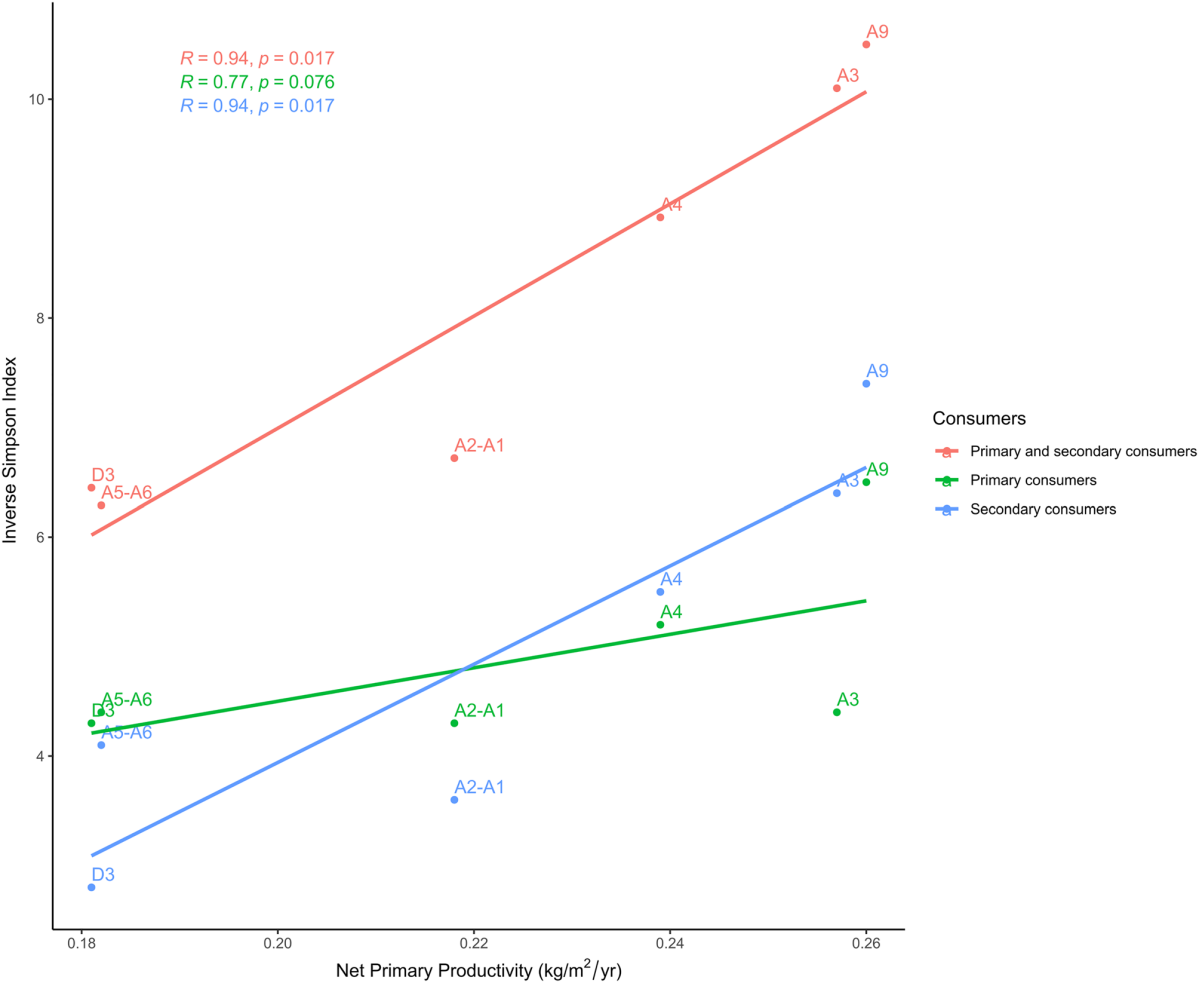
Linear correlation between Net Primary Productivity and the Inverse Simpson Index computed with the Minimum Number of Individuals of primary, secondary, and primary and secondary consumer species together recovered from the Fumane archaeological units. Labels show the archaeological unit of each dot, the correlation coefficient (R) and the p-value (p) of each correlation according to Spearman’s rank correlation test.

NPP fluctuations did not affect significantly herbivore diversity in Fumane (p-value 0.076), but it did the carnivore richness (p-value 0.017) and the whole mammalian diversity (p-value 0.017) recovered in each archaeological unit (Fig. 8). The NPP results suggest that fluctuations might have affected the species richness surrounding Fumane, so the drop-in ecosystem productivity during the Protoaurignacian likely affected, not only the diet breadth of the first modern humans but also the trophic pressure exerted by secondary consumers.

## Discussion

### Palaeoeconomic interpretation of Fumane Protoaurignacian

New insight into the subsistence strategies adopted by early modern human groups that occupied Fumane has been developed through the taphonomic and archaeozoological study of the complete macrofaunal assemblage of units A2–A1 and D3 located outside the cave. The data provide details about the palaeoeconomic behaviour of the different human groups that made the Protoaurignacian and late Protoaurignacian technocomplexes identified in those units. Altogether these new data expand our understanding of the activities carried out at the site, human mobility patterns and the types of environments that surrounded Fumane when AMH occupied the cave (Table S2).

The chronometric dates for the site (Table S6) cover the early Upper Palaeolithic in the Prealpine region between 42,000 and 37,750 cal BP, namely from GS11 to the end of GI9 and prove that modern humans were in Fumane during both stadial and interstadial times. Specifically, human occupations in unit D3 took place during H4. The new dates for the preceding Uluzzian archaeological unit A3, between 44,500 to 41,100 cal BP, which coincides with interstadial phases from GI11 to GI10, confirm previous radiocarbon dates undertaken only on charcoal^89^. The earlier published subsistence patterns indicate that humans targeted red deer and ibex, as well as giant deer, roe deer, bison and chamois of all age classes^66,158^. Compared to the Mousterian, the Uluzzian assemblage records a cooling of climatic conditions correlated with modifications in the game that was hunted comparable to those of the Protoaurignacian. Wolf, fox and brown bear were exploited as well^158^.

However, occupation of unit A2–A1 was more intensive than during D3, likely caused by the less favourable conditions during H4. After humans left the cave, carnivores hardly scavenged the human leftovers.

Protoaurignacian groups mainly exploited prime-age ibex and chamois, followed by red deer. Although a similar diet breath exists between both units, there is a reduction in red deer individuals in the D3 unit, likely caused by forest reduction which is broadly consistent with evidence from the environmental proxies at the site^163,164^. Humans exploited the montane areas surrounding the site, where both ibex and chamois dwelled, as well as forest species like cervids, and likely large bovines that could have occupied wooded areas. Interestingly, the exploitation of large bovines seems was relatively limited, although the protein yield provided by these large animals might have been significant for the human diet.

The proximity of the ibex kills might well explain the complete transport of this prey to the site. Various butchering activities related to the consumption and processing of the carcasses were carried out at the cave, as revealed by the different kinds of anthropic cut marks and evidence of green bone breakage. In addition, traces of wolf and fox fur preparation were attested, thus confirming human interest in these and other carnivores such as lynx^165^.

The mortality profiles reveal a profitable prey selection of prime-age individuals with significant, but non-majority representation of juveniles. Some of them were infantile ibex individuals that reveal late spring/summer hunting episodes, likely because humans were massacring female herds with young.

### Biodiversity in Italy during the Protoaurignacian

NPP usually correlates with the whole gamut of mammalian biodiversity in both extant and extinct ecosystems^166^, which is driven by the flow of energy through trophic levels. Food chain length increases along gradients of NPP^167^, so a higher NPP is not only associated with a greater abundance of herbivores, but also with the frequency of secondary consumers^166^. In this study, the results have revealed that the NPP is more correlated with the diversity of secondary than of primary consumers, which is probably because herbivore remains were mainly accumulated by humans and, therefore, are biased by hunting choices/preferences. In any case, the results show that the productivity-diversity relationships explain the mammalian richness recorded through the different Fumane units. Therefore, climatic conditions during the Protoaurignacian occupation reduced the base of the food chain (i.e., NPP) and, as a consequence, mammalian diversity and abundance were likely constrained. Under these ecological conditions before and during Heinrich Event 4 (H4), the resource availability for secondary consumer species decreased and, consequently, the diversity of secondary consumers also came down. Such a scenario is documented for the Protoaurignacian of Fumane, which suggests that early modern humans successfully coped with severe environmental conditions that had negatively affected the productivity and diversity of the ecosystem. This is also seen in the micromammalian assemblages of these units that indicate a slight decrease in woodland formations^163^. Except for the Mousterian A5–A6 unit, in the Protoaurignacian units, the NPP was significantly lower than in the other archaeological units of Fumane (Fig. 7).

**Figure 9:**
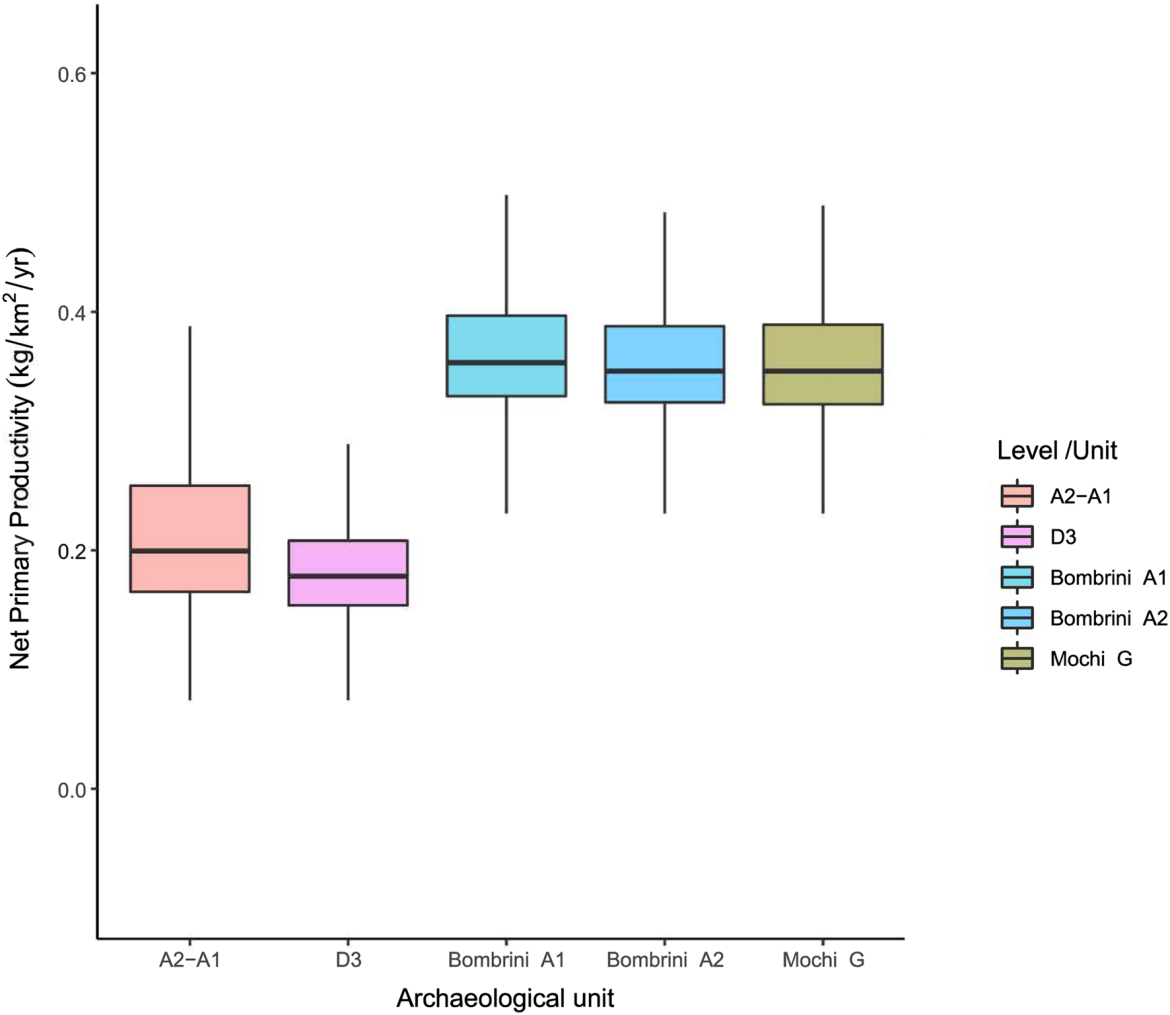
Box and whisker plot with the estimated NPP in different Protoaurignacian units of Italy.

If we want to evaluate the NPP in the Italian Peninsula at the time of arrival of the Protoaurignacian modern humans, in terms of a similar temporal span, technological culture and ecological setting, only Riparo Bombrini^8,168–171^ and Riparo Mochi^42,172,173^ in the Liguria region are comparable. Radiocarbon dates from Riparo Bombrini and Riparo Mochi provide some of the oldest evidence for the Protoaurignacian^42^, underscoring Liguria as one of the earliest areas occupied by modern humans in Western Europe. The appearance of the Protoaurignacian groups in these sites occurred during a phase of climatic degradation, with a sparsely forested environment, marked by the presence of plant taxa indicative of steppe-like conditions^168^, which placed human diffusion in Liguria to just after GI 10, dated to around 41.5 ky cal BP and extending through the climatic instability associated with Heinrich Event 4. In Bombrini, the macrofauna selected by humans reveal evidence of the palaeoenvironmental conditions. The high proportions of caprids, bovines, and red deer and the presence of equid species and rhinos would indicate cold conditions with more open habitats and patchy woods. The presence of horses and rhinos suggests that the sea level at that time was low enough to provide open plains in front of the Balzi Rossi^168,169^. The NPP estimated for Bombrini A1 and A2 and Mochi Level G show similar and stable values. However, the NPP is significantly lower in the D3 and A2–A1 units of Fumane (Fig. 9), probably because of the sensitivity of the Prealpine area to MIS3 climatic fluctuations. Recent palaeoclimatic reconstructions show that Eurosiberian regions experienced significant environmental changes during the Middle to Upper Palaeolithic transition^174^, while, in contrast, Mediterranean areas were much more stable^175^. For the first time, with available data, the present study preliminary shows the effects of these climatic fluctuations on the base of the food chain (NPP) and its significant correlation with the herbivore species diversity recorded in the Protoaurignacian Italian sites.

### Diversity in the productivity of European Protoaurignacian sites

**Figure 10:**
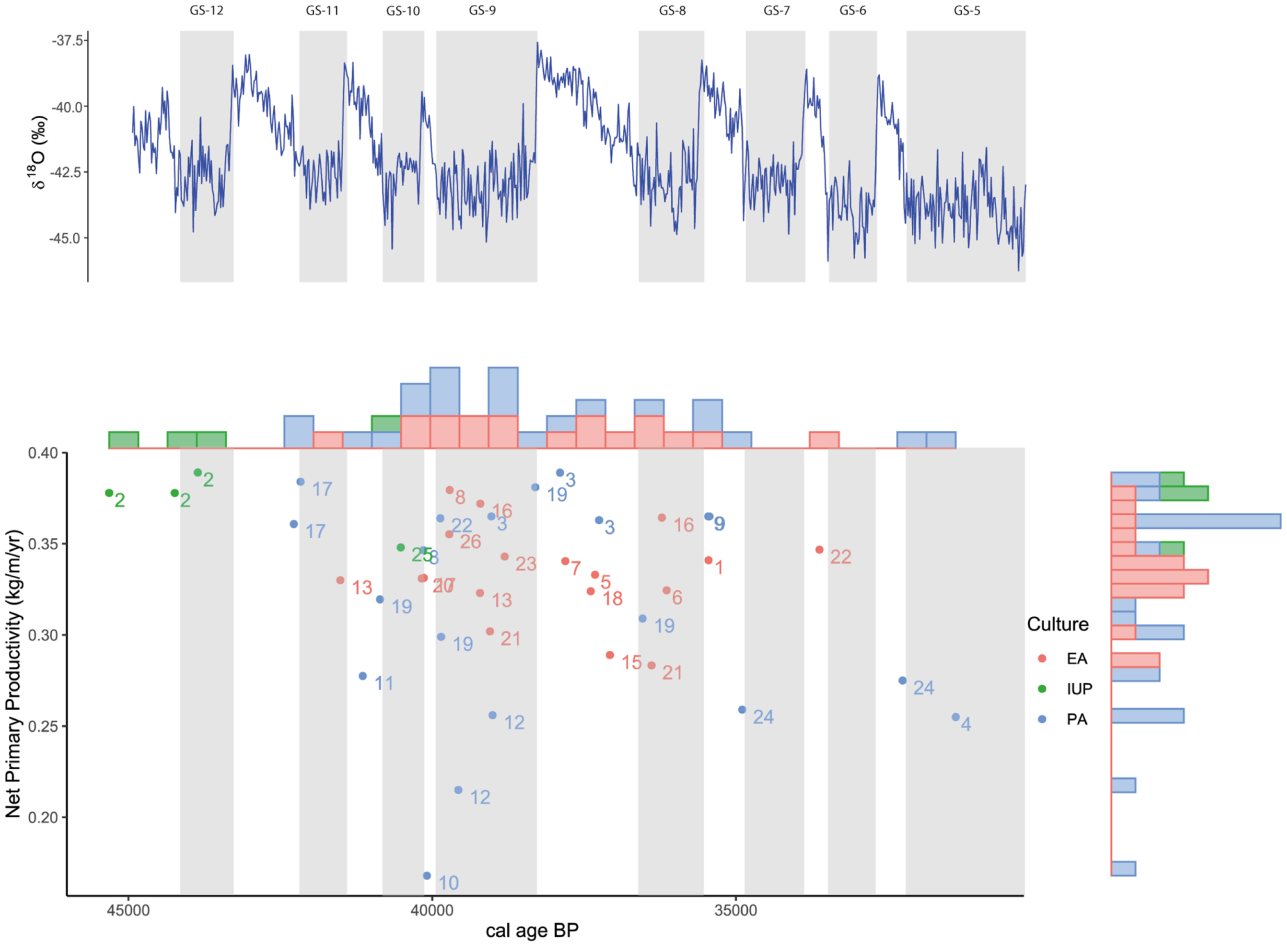
Temporal evolution of the Net Primary Productivity in each archaeological site with Initial Upper Palaeolithic (IUP), Early (EA) and Protoaurignacian (PA) assemblages. Vertical shaded bars indicate the stadial phases. 1. Aitzbitarte III, 2. Bacho Kiro, 3. Bombrini, 4. Cala, 5. Castanet, 6. Cova Foradada, 7. Cova Gran, 8. Covalejos, 9. Ekain, 10. El Cuco, 11. Esquicho-Grapaou, 12. Fumane, 13. Geißenklösterle, 14. Grotte du Renne, 15. Hohle Fels, 16. Istállóskő, 17. Isturitz, 18. La Quina, 19. Labeko Koba, 20. Lapa do Picareiro, 21. Les Cottes, 22. Mochi, 23. Roc de Combe, 24. Siuren I, 25. Temnata, 26. Terrases de la Riera dels Canyars (see Tables S3, S4, S5, S6 and S7 for details).

It has been argued that peopling of Europe by modern humans could be contingent on the environmental conditions^174^. Thus, it has been proposed that the stadial-interstadial cycles of the MIS3 generated a Neanderthal demographic crisis or even vacuum in some regions, which favoured the rapid spread of modern humans during the interstadial phases^176^. However, recent studies suggest that modern humans also spread through Europe during cold stadial conditions^2^. This idea is reinforced by the Protoaurignacian occupations of Fumane, where the arrival of modern humans was coeval with harsh environmental circumstances even including occupation of the site during the Heinrich Event 4. However, this association between the arrival of modern human groups and severe environmental conditions cannot be extended to all European sites or regions (Fig. 1). Thus, the Initial Upper Palaeolithic technology recovered in Bacho Kiro and Temnata is associated with a high NPP (Fig. 9, Table S7). Therefore, the early arrival and dispersal of modern humans through the Danube basin might have been motivated by the high productivity of the ecosystems in this region that served as a major migration route. In this regard, despite the European regions experiencing significant NPP fluctuations during the late MIS 3 (Fig. S5), the Protoaurignacian and Early Aurignacian occupations were mainly located in areas and periods of relatively high NPP (Fig. 9). These results reinforce the idea that ecosystem productivity was an important factor in the rapid dispersals of modern humans. Nevertheless, dispersal waves of human populations with Proto- and Early Aurignacian technologies also show an increasing variability in the NPP through time (Fig. 9). In this way, the Protoaurignacian levels of Fumane suggest that modern humans also inhabited and exploited ecosystems with low productivity (Fig. 10) similar to those exploited by Neanderthals during the Mousterian occupations of A5 + A6 and A6, coincident also with a stadial phase (GS12). Therefore, the spread of our species in Europe occurred under a wide range of climatic and environmental conditions, which reinforces the previously proposed environmental flexibility of modern humans^2^.

## Conclusions

In summary, the data obtained in this research have provided a wider knowledge about the earliest modern humans’ presence in Italy. Fumane has been proven to be a significant site to study the organisation of the Protoaurignacian human groups in the Prealpine region. The archaeozoological data of both units reveal evidence of subsistence and landscape changes in the vicinity of Fumane. These changes could have been caused by the severe condition during H4 when the D3 unit was accumulated. Human occupations were probably more sporadic and, due to the vegetation cover reduction, focused on the montane-adapted animals located in the vicinity of the cave. Evaluating the subsistence strategies adopted by those groups by comparing them with the Net Primary Productivity at that time, has helped to understand how those groups adapted their strategies and also reveal the resilience of our ancestors to cope with different ecosystems and stadial/interstadial phases in their wave of migration from the Near East to the western part of the continent. The low productivity of particular ecosystems did not stop the rapid migration of the Proto- and Early Aurignacian technocomplexes. In effect, the settlement of those groups was, mainly, in areas where high NPP is proven which might explain their successful advance and colonization of the continent.

## Supplementary Information

Supplementary Information.

Supplementary Tables.

## Data Availability

The datasets generated and/or analysed during the current study are available in the Supplementary Information.

## References

[1] Nigst PR (2014). Early modern human settlement of Europe north of the alps occurred 43,500 years ago in a cold steppe-type environment. Proc. Natl. Acad. Sci. U.S.A..

[2] Pederzani S (2021). Subarctic climate for the earliest *Homo sapiens* in Europe. Sci. Adv..

[3] Shao Y (2021). Human-existence probability of the Aurignacian techno-complex under extreme climate conditions. Quat. Sci. Rev..

[4] Slimak L (2022). Modern human incursion into Neanderthal territories 54,000 years ago at Mandrin, France. Sci. Adv..

[5] Fewlass H (2020). A 14C chronology for the Middle to Upper Palaeolithic transition at Bacho Kiro Cave, Bulgaria. Nat. Ecol. Evol..

[6] Hublin JJ (2020). Initial Upper Palaeolithic *Homo sapiens* from Bacho Kiro Cave, Bulgaria. Nature.

[7] Benazzi S (2011). Early dispersal of modern humans in Europe and implications for Neanderthal behaviour. Nature.

[8] Benazzi S (2015). The makers of the Protoaurignacian and implications for Neandertal extinction. Science.

[9] Cortés-Sánchez M (2019). An early Aurignacian arrival in southwestern Europe. Nat. Ecol. Evol..

[10] Vidal-Cordasco M, Ocio D, Hickler T, Marín-Arroyo AB (2022). Publisher Correction: Ecosystem productivity affected the spatiotemporal disappearance of Neanderthals in Iberia. Nat. Ecol. Evol..

[11] Fu Q (2015). An early modern human from Romania with a recent Neanderthal ancestor. Nature.

[12] Wood RE (2014). The chronology of the earliest Upper Palaeolithic in northern Iberia: New insights from L’Arbreda, Labeko Koba and La Viña. J. Hum. Evol..

[13] Hublin JJ (2015). The modern human colonization of western Eurasia: When and where?. Quat. Sci. Rev..

[14] Hublin JJ (2012). The earliest modern human colonization of Europe. Proc. Natl. Acad. Sci. U.S.A..

[15] Marín-Arroyo AB, Sanz-Royo A (2022). What Neanderthals and AMH ate: Reassessment of the subsistence across the Middle-Upper Palaeolithic transition in the Vasco-Cantabrian region of SW Europe. J. Quat. Sci..

[16] Semal P (2009). New data on the late Neandertals: Direct dating of the Belgian Spy fossils. Am. J. Phys. Anthropol..

[17] Welker F (2016). Palaeoproteomic evidence identifies archaic hominins associated with the Châtelperronian at the Grotte du Renne. Proc. Natl. Acad. Sci..

[18] Zilhão J (2006). Chronostratigraphy of the Middle-to-Upper Paleolithic transition in the Iberian Peninsula. Pyrenae Rev. Prehist. i Antig. la Mediter. Occident..

[19] Vanhaeren M, d’Errico F (2006). Aurignacian ethno-linguistic geography of Europe revealed by personal ornaments. J. Archaeol. Sci..

[20] Higham T (2012). Testing models for the beginnings of the Aurignacian and the advent of figurative art and music: The radiocarbon chronology of Geißenklösterle. J. Hum. Evol..

[21] Broglio A (2009). L’art aurignacien dans la décoration de la Grotte de Fumane. Anthropologie.

[22] Conard NJ (2003). Palaeolithic ivory sculptures from southwestern Germany and the origins of figurative art. Nature.

[23] Conard NJ (2009). A female figurine from the basal Aurignacian of Hohle Fels Cave in southwestern Germany. Nature.

[24] Bourrillon R (2018). A new Aurignacian engraving from Abri Blanchard, France: Implications for understanding Aurignacian graphic expression in Western and Central Europe. Quat. Int..

[25] Tejero JM, Grimaldi S (2015). Assessing bone and antler exploitation at Riparo Mochi (Balzi Rossi, Italy): Implications for the characterization of the Aurignacian in South-western Europe. J. Archaeol. Sci..

[26] Tejero JM, Langley MC (2017). Spanish aurignacian projectile points: An example of the First European Paleolithic hunting weapons in osseous materials. Osseous Projectile Weaponry Towards an Understanding of Pleistocene Cultural Variability.

[27] Kitagawa K, Conard NJ (2020). Split-based points from the Swabian Jura highlight Aurignacian regional signatures. PLoS ONE.

[28] Floss H, Hoyer CT, Heckel C, Tartar É (2015). The Aurignacian in Southern Burgundy. Palethnologie.

[29] Tartar É (2015). Origin and development of Aurignacian Osseous Technology in Western Europe: A review of current knowledge. Palethnologie.

[30] Bar-Yosef O, Conard NJ (2006). Neanderthals and modern humans: A different interpretation. When Neanderthals and Modern Humans Met.

[31] Flas, D. Les pointes foliacées et les changements techniques autour de la transitiondu Paléolithiquemoyen au supérieur dans leNord-Ouest de l’Europe. In (eds Toussaint, M. & Di Modica, K. S. P.) 261–276 (ERAUL 128, 2011).

[32] Nigst PR (2012). The Early Upper Palaeolithic of the Middle Danube Region—Human Evolution.

[33] Tsanova, T. *Les débuts du Paléolithique supérieur dans l’Est des Balkans. Réflexion à partir de l’étude taphonomique et techno-économique des ensembles lithiques des sites de Bacho Kiro (couche 11), Temnata (couches VI et 4) et Kozarnika (niveau VII)* (2008).

[34] Bosch MD (2015). New chronology for Ksâr ’Akil (Lebanon) supports Levantine route of modern human dispersal into Europe. Proc. Natl. Acad. Sci. U.S.A..

[35] Chu W (2022). Aurignacian dynamics in Southeastern Europe based on spatial analysis, sediment geochemistry, raw materials, lithic analysis, and use-wear from Românești-Dumbrăvița. Sci. Rep..

[36] Conard NJ (2002). The timing of cultural innovations and the dispersal of modern humans in Europe. Terra Nostra.

[37] Davies, W. *Re-evaluating the Aurignacian as an Expression of Modern Human Mobility and Dispersal*. (eds. Mellars P, Boyle K, Bar-Yosef O, S. C.) (2001).

[38] Mellars P (2006). A new radiocarbon revolution and the dispersal of modern humans in Eurasia. Nature.

[39] Mellars P (2006). Archeology and the dispersal of modern humans in Europe: Deconstructing the ‘Aurignacian’. Evol. Anthropol..

[40] Szmidt CC, Normand C, Burr GS, Hodgins GWL, LaMotta S (2010). AMS 14C dating the Protoaurignacian/Early Aurignacian of Isturitz, France. Implications for Neanderthal-modern human interaction and the timing of technical and cultural innovations in Europe. J. Archaeol. Sci..

[41] Conard NJ, Bolus M (2003). Radiocarbon dating the appearance of modern humans and timing of cultural innovations in Europe: New results and new challenges. J. Hum. Evol..

[42] Douka K, Grimaldi S, Boschian G, del Lucchese A, Higham TFG (2012). A new chronostratigraphic framework for the Upper Palaeolithic of Riparo Mochi (Italy). J. Hum. Evol..

[43] Davies W, Hedges REM (2009). Dating a type site: Fitting Szeleta Cave into its regional chronometric context. Praehistoria.

[44] Davies W, White D, Lewis M, Stringer C (2015). Evaluating the transitional mosaic: Frameworks of change from Neanderthals to Homo sapiens in eastern Europe. Quat. Sci. Rev..

[45] Marín-Arroyo AB (2018). Chronological reassessment of the Middle to Upper Paleolithic transition and Early Upper Paleolithic cultures in Cantabrian Spain. PLoS ONE.

[46] Barshay-Szmidt C, Normand C, Flas D, Soulier MC (2018). Radiocarbon dating the Aurignacian sequence at Isturitz (France): Implications for the timing and development of the Protoaurignacian and Early Aurignacian in western Europe. J. Archaeol. Sci. Rep..

[47] Wood R (2018). El Castillo (Cantabria, northern Iberia) and the Transitional Aurignacian: Using radiocarbon dating to assess site taphonomy. Quat. Int..

[48] Chu W (2018). The danube corridor hypothesis and the carpathian basin: Geological, environmental and archaeological approaches to characterizing aurignacian dynamics. J. World Prehist..

[49] Falcucci A, Conard NJ, Peresani M (2020). Breaking through the aquitaine frame: A re-evaluation on the significance of regional variants during the Aurignacian as seen from a key record in southern Europe. J. Anthropol. Sci..

[50] Banks WE, d’Errico F, Zilhão J (2013). Human-climate interaction during the Early Upper Paleolithic: Testing the hypothesis of an adaptive shift between the Proto-Aurignacian and the Early Aurignacian. J. Hum. Evol..

[51] Badino F (2020). An overview of Alpine and Mediterranean palaeogeography, terrestrial ecosystems and climate history during MIS 3 with focus on the Middle to Upper Palaeolithic transition. Quat. Int..

[52] Bataille G, Conard NJ (2018). Blade and bladelet production at Hohle Fels Cave, AH IV in the Swabian Jura and its importance for characterizing the technological variability of the Aurignacian in Central Europe. PLoS ONE.

[53] Higham T, Wood R, Moreau L, Conard N, Ramsey CB (2013). Comments on ‘Human-climate interaction during the early Upper Paleolithic: Testing the hypothesis of an adaptive shift between the Proto-Aurignacian and the Early Aurignacian’ by Banks et al.. J. Hum. Evol..

[54] Teyssandier N, Zilhão J (2018). On the entity and antiquity of the Aurignacian at Willendorf (Austria): Implications for modern human emergence in Europe. J. Paleolit. Archaeol..

[55] Discamps E, Jaubert J, Bachellerie F (2011). Human choices and environmental constraints: Deciphering the variability of large game procurement from Mousterian to Aurignacian times (MIS 5–3) in southwestern France. Quat. Sci. Rev..

[56] Kuhn SL, Stiner MC (2006). What’s a mother to do? The division of labor among Neandertals and modern humans in Eurasia. Curr. Anthropol..

[57] Starkovich BM (2012). Intensification of small game resources at Klissoura Cave 1 (Peloponnese, Greece) from the Middle Paleolithic to Mesolithic. Quat. Int..

[58] Stiner M (2009). Prey choice, site occupation intensity & economic diversity in the Middle–early Upper Palaeolithic at the Üçağizli Caves. Turkey. Before Farm..

[59] de los Terreros JY, Gómez-Castanedo A, Aramendi-Picado J, Montes-Barquín R, Sanguino-González J (2016). Neanderthal and*Homo sapiens*subsistence strategies in the Cantabrian region of northern Spain. Archaeol. Anthropol. Sci..

[60] Bertacchi A, Starkovich BM, Conard NJ (2021). The Zooarchaeology of Sirgenstein Cave: A Middle and Upper Paleolithic site in the Swabian Jura, SW Germany. J. Paleolit. Archaeol..

[61] Boscato P, Crezzini J (2012). Middle-Upper Palaeolithic transition in Southern Italy: Uluzzian macromammals from Grotta del Cavallo (Apulia). Quat. Int..

[62] Grayson DK, Delpech F (2008). The large mammals of Roc de Combe (Lot, France): The Châtelperronian and Aurignacian assemblages. J. Anthropol. Archaeol..

[63] Morin E (2019). New evidence of broader diets for archaic Homo populations in the northwestern Mediterranean. Sci. Adv..

[64] Münzel S, Conard NJ (2004). Change and continuity in subsistence during the Middle and Upper Palaeolithic in the Ach Valley of Swabia (South-west Germany). Int. J. Osteoarchaeol..

[65] Rendu W (2019). Subsistence strategy changes during the Middle to Upper Paleolithic transition reveals specific adaptations of Human Populations to their environment. Sci. Rep..

[66] Romandini M (2020). Macromammal and bird assemblages across the late Middle to Upper Palaeolithic transition in Italy: An extended zooarchaeological review. Quat. Int..

[67] Starkovich BM (2017). Paleolithic subsistence strategies and changes in site use at Klissoura Cave 1 (Peloponnese, Greece). J. Hum. Evol..

[68] Peresani M (2022). Inspecting human evolution from a cave Late Neanderthals and early sapiens at Grotta di Fumane: Present state and outlook. J. Anthropol. Sci..

[69] Jarvis, A. *et al*. *Hole-Filled Seamless SRTM Data V4*. https://srtm.csi.cgiar.org (International Centre for Tropical Agriculture (CIAT), 2008).

[70] Delpiano D, Heasley K, Peresani M (2018). Assessing Neanderthal land use and lithic raw material management in discoid technology. J. Anthropol. Sci..

[71] Marcazzan D, Ligouis B, Duches R, Conard NJ (2022). Middle and Upper Paleolithic occupations of Fumane Cave (Italy): A geoarchaeological investigation of the anthropogenic features. J. Antropol. Sci..

[72] Douka K (2014). On the chronology of the Uluzzian. J. Hum. Evol..

[73] Peresani M, Cristiani E, Romandini M (2016). The Uluzzian technology of Grotta di Fumane and its implication for reconstructing cultural dynamics in the Middle-Upper Palaeolithic transition of Western Eurasia. J. Hum. Evol..

[74] Peresani M, Bertola S, Delpiano D, Benazzi S, Romandini M (2019). The Uluzzian in the north of Italy: Insights around the new evidence at Riparo Broion. Archaeol. Anthropol. Sci..

[75] Aleo A, Duches R, Falcucci A, Rots V, Peresani M (2021). Scraping hide in the early Upper Paleolithic: Insights into the life and function of the Protoaurignacian endscrapers at Fumane Cave. Archaeol. Anthropol. Sci..

[76] Falcucci A, Conard NJ, Peresani M (2017). A critical assessment of the Protoaurignacian lithic technology at Fumane Cave and its implications for the definition of the earliest Aurignacian. PLoS ONE.

[77] Falcucci A, Peresani M (2019). A pre-Heinrich Event 3 assemblage at Fumane Cave and its contribution for understanding the beginning of the Gravettian in Italy. Quartär.

[78] Higham T (2011). European Middle and Upper Palaeolithic radiocarbon dates are often older than they look. Antiquity.

[79] Higham T (2009). Problems with radiocarbon dating the Middle to Upper Palaeolithic transition in Italy. Quat. Sci. Rev..

[80] Cassoli PF, Tagliacozzo A (1994). Considerazioni paleontologiche, paleoecologiche e archeologiche sui micromammiferi e gli uccelli dei livelli del Pleistocene Superiore del Riparo di Fumane (Vr) (Scavi 1988–91). Bollettino del Museo Civico di Storia Naturale di Verona.

[81] Broglio A, De Stefani M, Tagliacozzo A, Gurioli F, Facciolo A, Vasilev SA (2006). Aurignacian dwelling structures, hunting strategies and seasonality in the Fumane Cave (Lessini Mountains). Kostenki and the Early Upper Paleolithic of Eurasia: General Trends, Local Developments.

[82] Bertola S, Djindjian F, Kozlowski JK, Bicho N (2009). Le territoire des chasseurs aurignaciens dans les Préalpes de la Vénétie: l’exemple de la Grotte de Fumane. Le Concept de Territoires dans le Paléolithique Supérieur Européen.

[83] Jéquier C, Livraghi A, Romandini M, Peresani M, Hutson JM (2018). Same but different: 20,000 years of bone retouchers from northern Italy. A diachronologic approach from neanderthals to anatomically modern humans. The Origins of Bone Tool Technologies.

[84] Marín-Arroyo AB (2009). A comparative study of analytic techniques for skeletal part profile interpretation at El Mirón Cave (Cantabria, Spain). Archaeofauna.

[85] Romandini, M. *Analisi archeozoologica, tafonomica, paleontologica e spaziale dei livelli Uluzziani e tardo-Musteriani della Grotta di Fumane (VR). Variazioni e continuità strategico-comportamentali umane in Italia Nord Occidentale: i casi di Grotta del Col della Stria*. Dip. di Biol. ed Evol. PhD thesis 505 (2012).

[86] Marean CW, Abe Y, Nilssen PJ, Stone EC (2001). Estimating the minimum number of skeletal elements (MNE) in zooarchaeology: A review and a new image-analysis GIS approach. Am. Antiq..

[87] Stiner MC (1990). The use of mortality patterns in archaeological studies of hominid predatory adaptations. J. Anthropol. Archaeol..

[88] Marín-Arroyo AB, Morales MRG (2009). Comportamiento económico de los últimos cazadores-recolectores y primeras evidencias de domesticación en el occidente de asturias. La cueva de mazaculos II. Trab. Prehist..

[89] Simpson EH (1949). Measurement of diversity. Nature.

[90] Magurran AE (1988). Ecological Diversity and Its Measurement.

[91] Marín-Arroyo AB (2009). The use of optimal foraging theory to estimate Late Glacial site catchment areas from a central place: The case of eastern Cantabria, Spain. J. Anthropol. Archaeol..

[92] Azorit C (2011). Guía para la determinación de la edad del ciervo ibérico (*Cervus elaphus* hispanicus) a través de su dentición: Revisión metodológica y técnicas de elección. An. la Real Acad. Ciencias Vet. Andalucía Orient..

[93] Mariezkurrena K (1983). Contribución al conocimiento del desarrollo de la dentición y el esqueleto poscraneal de *Cervus elaphus*. Munibe.

[94] Tomé C, Vigne JD (2003). Roe deer (*Capreolus capreolus*) age at death estimates: New methods and modern reference data for tooth eruption and wear, and for epiphyseal fusion. Archaeofauna.

[95] Couturier MAJ (1962). Le bouquetin des Alpes: Capra aegagrus ibex ibex L..

[96] Habermehl K-H (1992). Die Altersbeurteilung beim weiblichen Steinwild (*Capra ibex ibex* L.) anhand der Skelettentwicklung. Anat. Histol. Embryol. J. Vet. Med. Ser. C.

[97] Pflieger RHP (1982). Le chamois, son identification et sa vie.

[98] Binford LR (1978). Nunamiut Etnoarchaeology.

[99] Metcalfe D, Jones KT (1988). A reconsideration of animal body-part utility indices. Am. Antiq..

[100] Morin E, Ready E, Clark JL, Speth JD (2013). Foraging goals and transport decisions in western Europe during the Paleolithic and Early Holocene. Zooarchaeology and Modern Human Origins. Vertebrate Paleobiology and Paleoanthropology.

[101] Lam YM, Chen X, Pearson OM (1999). Intertaxonomic variability in patterns of bone density and the differential representation of bovid, cervid, and equid elements in the archaeological record. Am. Antiq..

[102] Morin E (2007). Fat composition and Nunamiut decision-making: A new look at the marrow and bone grease indices. J. Archaeol. Sci..

[103] Marín-Arroyo AB, Ocio D (2018). Disentangling faunal skeletal profiles. A new probabilistic framework. Hist. Biol..

[104] Rogers AR (2000). On the value of soft bones in faunal analysis. J. Archaeol. Sci..

[105] Rogers AR (2000). Analysis of bone counts by maximum likelihood. J. Archaeol. Sci..

[106] Marín-Arroyo, A. B., Ocio, D., Vidal-Cordasco, M. & Vettese, D. *BaSkePro: Bayesian Model to Archaeological Faunal Skeletal Profiles*. R package version 0.1.0. https://CRAN.R-project.org/package=BaSkePro (2022).

[107] Binford LR (1981). Bones Ancient Men and Modern Myths.

[108] Galán AB, Domínguez-Rodrigo M (2013). An experimental study of the anatomical distribution of cut marks created by filleting and disarticulation on long bone ends. Archaeometry.

[109] Nilssen PJ (2000). An Actualistic Butchery Study in South Africa and Its Implications for Reconstructing Hominid Strategies of Carcass Acquisition and Butchery in the Upper Pleistocene and Plio-Pleistocene.

[110] Capaldo SD, Blumenschine RJ (1994). A quantitative diagnosis of notches made by hammerstone percussion and carnivore gnawing on bovid long bones. Am. Antiq..

[111] Pickering TR, Egeland CP (2006). Experimental patterns of hammerstone percussion damage on bones: Implications for inferences of carcass processing by humans. J. Archaeol. Sci..

[112] Bunn HT (1981). Archaeological evidence for meat-eating by Plio-Pleistocene hominids from Koobi Fora and Olduvai Gorge. Nature.

[113] Villa P, Mahieu E (1991). Breakage patterns of human long bones. J. Hum. Evol..

[114] Blumenschine RJ, Selvaggio MM (1988). Percussion marks on bone surfaces as a new diagnostic of hominid behaviour. Nature.

[115] Galán AB, Rodríguez M, de Juana S, Domínguez-Rodrigo M (2009). A new experimental study on percussion marks and notches and their bearing on the interpretation of hammerstone-broken faunal assemblages. J. Archaeol. Sci..

[116] Pickering TR (2013). Taphonomy of ungulate ribs and the consumption of meat and bone by 1.2-million-year-old hominins at Olduvai Gorge, Tanzania. J. Archaeol. Sci..

[117] Vettese D (2020). Towards an understanding of hominin marrow extraction strategies: A proposal for a percussion mark terminology. Archaeol. Anthropol. Sci..

[118] Vettese D (2021). A way to break bones? The weight of intuitiveness. PLoS ONE.

[119] Coil R, Yezzi-Woodley K, Tappen M (2020). Comparisons of impact flakes derived from hyena and hammerstone long bone breakage. J. Archaeol. Sci..

[120] Stiner MC, Kuhn SL, Weiner S, Bar-Yosef O (1995). Differential burning, recrystallization, and fragmentation of archaeological bone. J. Archaeol. Sci..

[121] Mallye JB (2012). The Mousterian bone retouchers of Noisetier Cave: Experimentation and identification of marks. J. Archaeol. Sci..

[122] Blumenschine RJ (1995). Percussion marks, tooth marks, and experimental determinations of the timing of hominid and carnivore access to long bones at FLK Zinjanthropus, Olduvai Gorge, Tanzania. J. Hum. Evol..

[123] Domínguez-Rodrigo M, Piqueras A (2003). The use of tooth pits to identify carnivore taxa in tooth-marked archaeofaunas and their relevance to reconstruct hominid carcass processing behaviours. J. Archaeol. Sci..

[124] Domínguez-Rodrigo M, Barba R (2006). New estimates of tooth mark and percussion mark frequencies at the FLK Zinj site: The carnivore-hominid-carnivore hypothesis falsified. J. Hum. Evol..

[125] Behrensmeyer AK (1978). Taphonomic and écologie information from bone weathering. Paleobiology.

[126] Fisher JW (1995). Bone surface modifications in zooarchaeology. J. Archaeol. Method Theory.

[127] Lyman RL (1994). Vertebrate Taphonomy.

[128] Shipman P (1981). Life History of a Fossil: An Introduction to Taphonomy and Paleoecology.

[129] Marín-Arroyo AB (2008). Archaeological implications of human-derived manganese coatings: A study of blackened bones in El Mirón Cave, Cantabrian Spain. J. Archaeol. Sci..

[130] Marín-Arroyo AB, Landete-Ruiz MD, Seva-Román R, Lewis MD (2014). Manganese coating of the Tabun faunal assemblage: Implications for modern human behaviour in the Levantine Middle Palaeolithic. Quat. Int..

[131] Blasco R, Rosell J, Fernández Peris J, Cáceres I, Vergès JM (2008). A new element of trampling: An experimental application on the Level XII faunal record of Bolomor Cave (Valencia, Spain). J. Archaeol. Sci..

[132] Domínguez-Rodrigo M, de Juana S, Galán AB, Rodríguez M (2009). A new protocol to differentiate trampling marks from butchery cut marks. J. Archaeol. Sci..

[133] Hickler T, Prentice IC, Smith B, Sykes MT, Zaehle S (2006). Implementing plant hydraulic architecture within the LPJ dynamic global vegetation model. Glob. Ecol. Biogeogr..

[134] Zampieri D (2000). Segmentation and linkage of the Lessini Mountains normal faults, Southern Alps, Italy. Tectonophysics.

[135] Castiglioni GB, Cremaschi M (1990). The loess deposits in the Lessini plateau. The Loess in Northern and Central Italy: A Loess Basin Between the Alps and the Mediterranean Region.

[136] Sauro U (1973). Il paesaggio degli alti Lessini. Studio Geomofologico.

[137] Castiglioni, G. B. *et al*. *Geomorphological Map of Po Plain, Scale 1:250,000* (1997).

[138] Fontana A, Mozzi P, Marchetti M (2014). Alluvial fans and megafans along the southern side of the Alps. Sediment. Geol..

[139] Holechek JL, Pieper RD, Herbel CH (1998). Range Management Principles and Practices.

[140] Imhoff ML (2004). Global patterns in human consumption of net primary production. Nature.

[141] Sitch S (2003). Evaluation of ecosystem dynamics, plant geography and terrestrial carbon cycling in the LPJ dynamic global vegetation model. Glob. Change Biol..

[142] Smith B (2014). Implications of incorporating N cycling and N limitations on primary production in an individual-based dynamic vegetation model. Biogeosciences.

[143] Githumbi EN (2018). Pollen, people and place: Multidisciplinary perspectives on ecosystem change at Amboseli, Kenya. Front. Earth Sci..

[144] Allen JRM (2020). Global vegetation patterns of the past 140,000 years. J. Biogeogr..

[145] Armstrong E, Hopcroft PO, Valdes PJ (2019). A simulated Northern Hemisphere terrestrial climate dataset for the past 60,000 years. Sci. Data.

[146] Harris I, Osborn TJ, Jones P, Lister D (2020). Version 4 of the CRU TS monthly high-resolution gridded multivariate climate dataset. Sci. Data.

[147] Adam M, Weitzel N, Rehfeld K (2021). Identifying global-scale patterns of vegetation change during the last deglaciation from paleoclimate networks. Paleoceanogr. Paleoclimatol..

[148] Beyer R, Krapp M, Manica A (2020). An empirical evaluation of bias correction methods for palaeoclimate simulations. Clim. Past.

[149] Lüthi D (2008). High-resolution carbon dioxide concentration record 650,000–800,000 years before present. Nature.

[150] Zobler, L. *A World Soil File for Global Climate Modelling. NASA Technical Memorandum 87802* (1986).

[151] Bronk Ramsey C (2009). Bayesian analysis of radiocarbon dates. Radiocarbon.

[152] Reimer PJ (2020). The IntCal20 northern hemisphere radiocarbon age calibration curve (0–55 cal kBP). Radiocarbon.

[153] Andersen KK (2006). The Greenland ice core chronology 2005, 15–42 ka. Part 1: Constructing the time scale. Quat. Sci. Rev..

[154] Svensson A (2008). A 60,000 year Greenland stratigraphic ice core chronology. Clim. Past.

[155] Rasmussen SO (2014). A stratigraphic framework for abrupt climatic changes during the Last Glacial period based on three synchronized Greenland ice-core records: Refining and extending the INTIMATE event stratigraphy. Quat. Sci. Rev..

[156] Higham T (2014). The timing and spatiotemporal patterning of Neanderthal disappearance. Nature.

[157] Romandini M, Nannini N, Tagliacozzo A, Peresani M (2014). The ungulate assemblage from layer A9 at Grotta di Fumane, Italy: A zooarchaeological contribution to the reconstruction of Neanderthal ecology. Quat. Int..

[158] Tagliacozzo A, Romandini M, Fiore I, Gala M, Peresani M, Clark J, Speth J (2013). 2013. Animal exploitation strategies during the Uluzzian at Grotta di Fumane (Verona, Italy). Zooarchaeology and Modern Human Origins. Vertebrate Paleobiology and Paleoanthropology.

[159] Terlato G, Livraghi A, Romandini M, Peresani M (2019). Large bovids on the Neanderthal menu: Exploitation of *Bison priscus* and *Bos primigenius* in northeastern Italy. J. Archaeol. Sci. Rep..

[160] Peresani M, Fiore I, Gala M, Romandini M, Tagliacozzo A (2011). Late Neandertals and the intentional removal of feathers as evidenced from bird bone taphonomy at Fumane Cave 44 ky B.P., Italy. Proc. Natl. Acad. Sci. U.S.A..

[161] Fiore I (2016). From feathers to food: Reconstructing the complete exploitation of avifaunal resources by Neanderthals at Fumane cave, unit A9. Quat. Int..

[162] Romandini M (2016). Neanderthal scraping and manual handling of raptors wing bones: Evidence from Fumane Cave. Experimental activities and comparison. Quat. Int..

[163] López-García JM, dalla Valle C, Cremaschi M, Peresani M (2015). Reconstruction of the Neanderthal and Modern Human landscape and climate from the Fumane cave sequence (Verona, Italy) using small-mammal assemblages. Quat. Sci. Rev..

[164] Maspero A (1998). Ricostruzione del paesaggio vegetale attorno alla grotta di Fumane durante il Paleolitico. Annu. Stor. della Valpolicella.

[165] Malerba, G. & Giacobini, G. Analisi delle tracce di macellazione in un sito Paleolitico. L’esempio del Riparo di Fumane (Valpolicella, Verona). In *Atti del: “I° Convegno Nazionale di Archeozoologia”, Rovigo 5–7 marzo 1993* 97–108 (1995).

[166] Fritz SA (2016). Twenty-million-year relationship between mammalian diversity and primary productivity. Proc. Natl. Acad. Sci..

[167] Morris RJ (2008). Community ecology: How green is the arctic Tundra?. Curr. Biol..

[168] Holt B (2019). The Middle-Upper Paleolithic transition in Northwest Italy: New evidence from Riparo Bombrini (Balzi Rossi, Liguria, Italy). Quat. Int..

[169] Pothier Bouchard G, Riel-Salvatore J, Negrino F, Buckley M (2020). Archaeozoological, taphonomic and ZooMS insights into the Protoaurignacian faunal record from Riparo Bombrini. Quat. Int..

[170] Riel-Salvatore J, Negrino F, Robinson E, Sellet F (2018). Proto-Aurignacian Lithic technology, mobility, and human niche construction: A case study from Riparo Bombrini, Italy. Lithic Technological Organization and Paleoenvironmental Change, Studies in Human Ecology and Adaptation.

[171] Riel-Salvatore J, Negrino F (2018). Human adaptations to climatic change in Liguria across the Middle-Upper Paleolithic transition. J. Quat. Sci..

[172] Grimaldi S, Porraz G, Santaniello F (2014). Raw material procurement and land use in the northern Mediterranean Arc: Insight from the first Proto-Aurignacian of Riparo Mochi (Balzi Rossi, Italy). Quartar.

[173] Alaique F, Malerba G, Cilli C, Giacobini G (2000). Risultati preliminari dell’analisi dei resti faunistici rinvenuti nei livelli del Paleolitico superiore di Riparo Mochi (Balzi Rossi): Scavi 1995–1996. Atti Del 2°Convegno Nazionale Di Archeozoologia (Asti, 1997).

[174] Staubwasser M (2018). Impact of climate change on the transition of Neanderthals to modern humans in Europe. Proc. Natl. Acad. Sci..

[175] Columbu A (2020). Speleothem record attests to stable environmental conditions during Neanderthal–modern human turnover in southern Italy. Nat. Ecol. Evol..

[176] Müller UC (2011). The role of climate in the spread of modern humans into Europe. Quat. Sci. Rev..

