# Supplementary material for: Subsistence of early anatomically modern humans in Europe as evidenced in the Protoaurignacian occupations of Fumane Cave, Italy
Source: Sci Rep. 2023 Mar 7;13:3788. doi: 10.1038/s41598-023-30059-3 (PMC9992387; doi:10.1038/s41598-023-30059-3)
Supplement: Supplementary file 1 — Supplementary Information. [file 41598_2023_30059_MOESM1_ESM.docx]

**SUPPLEMENTARY INFORMATION 1**

**Subsistence of early anatomically modern humans in Europe: the Protoaurignacian occupations at Fumane Cave, Italy**

Ana B. Marín-Arroyo^1*,^ Gabriele Terlato^1*^, Marco Vidal-Cordasco^1^, Marco Peresani^2,3^

^1^ Grupo de I+D+i EVOADAPTA (Evolución Humana y Adaptaciones durante la Prehistoria). Dpto. Ciencias Históricas. Universidad de Cantabria. Av/Los Castros 44, 39005 Santander, Spain.

^2^ Dipartimento di Studi Umanistici, Sezione di Scienze Preistoriche e Antropologiche, Università di Ferrara, Ferrara, Italy.

^2^ Istituto di Geologia Ambientale e Geoingegneria, Consiglio Nazionale delle Ricerche, Milano, Italy.

*Corresponding authors: [anabelen.marin@unican.es](mailto:anabelen.marin@unican.es) and [gabriele.terlato@unican.es](mailto:gabriele.terlato@unican.es)


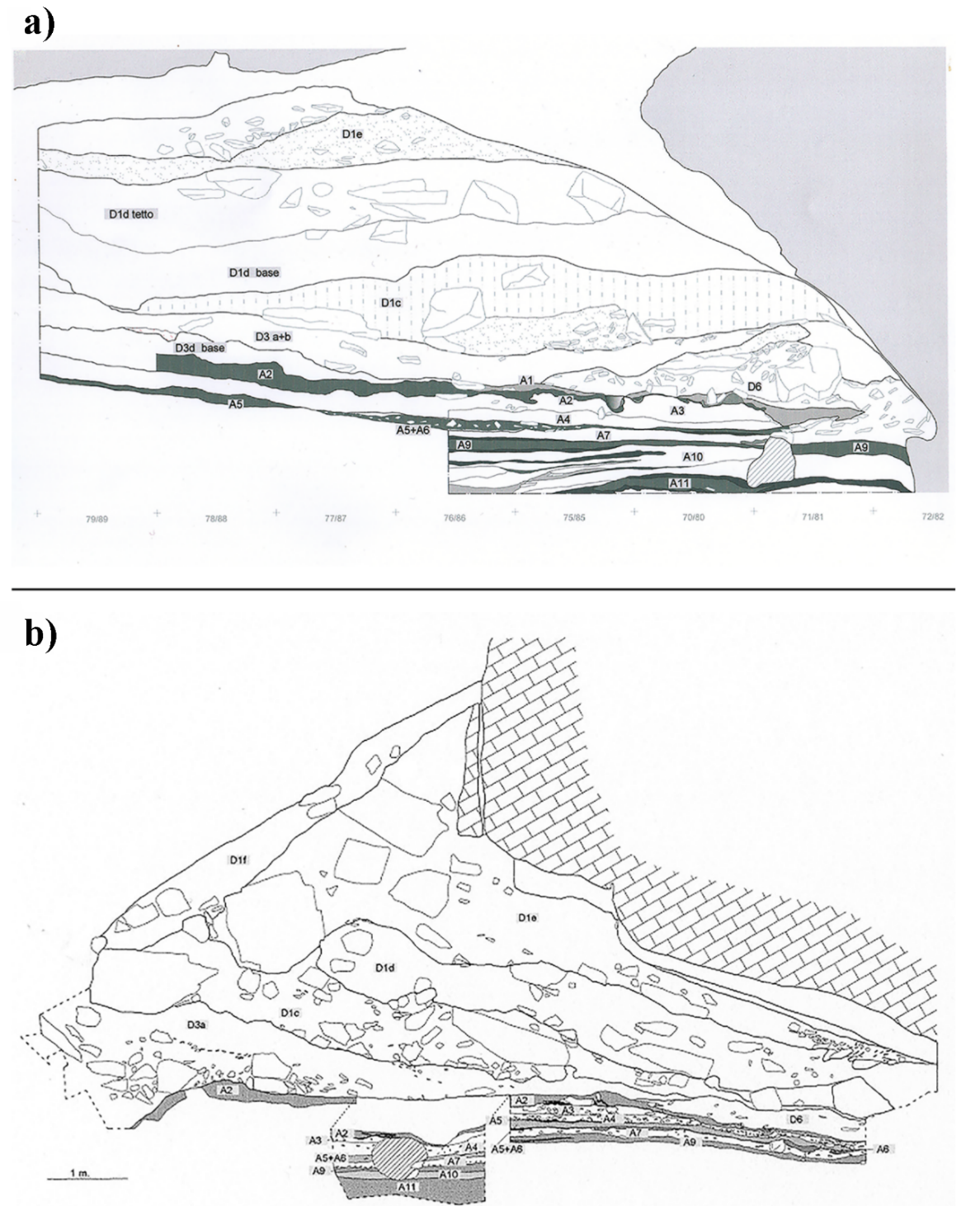


**Figure S1. a)** Cross section view showing the units corresponding to the late Mousterian (A11-A4), Uluzzian (A3), early Protoaurignacian (A2–A1), late Protoaurignacian (D6–D3a+b), and the stratigraphic complex D1; **b**) Cross sagittal view showing the succession of layers described above (Section **a** by M. Peresani and S. Muratori; section **b** by M. Cremaschi and M. Peresani, redrawn by S. Muratori).


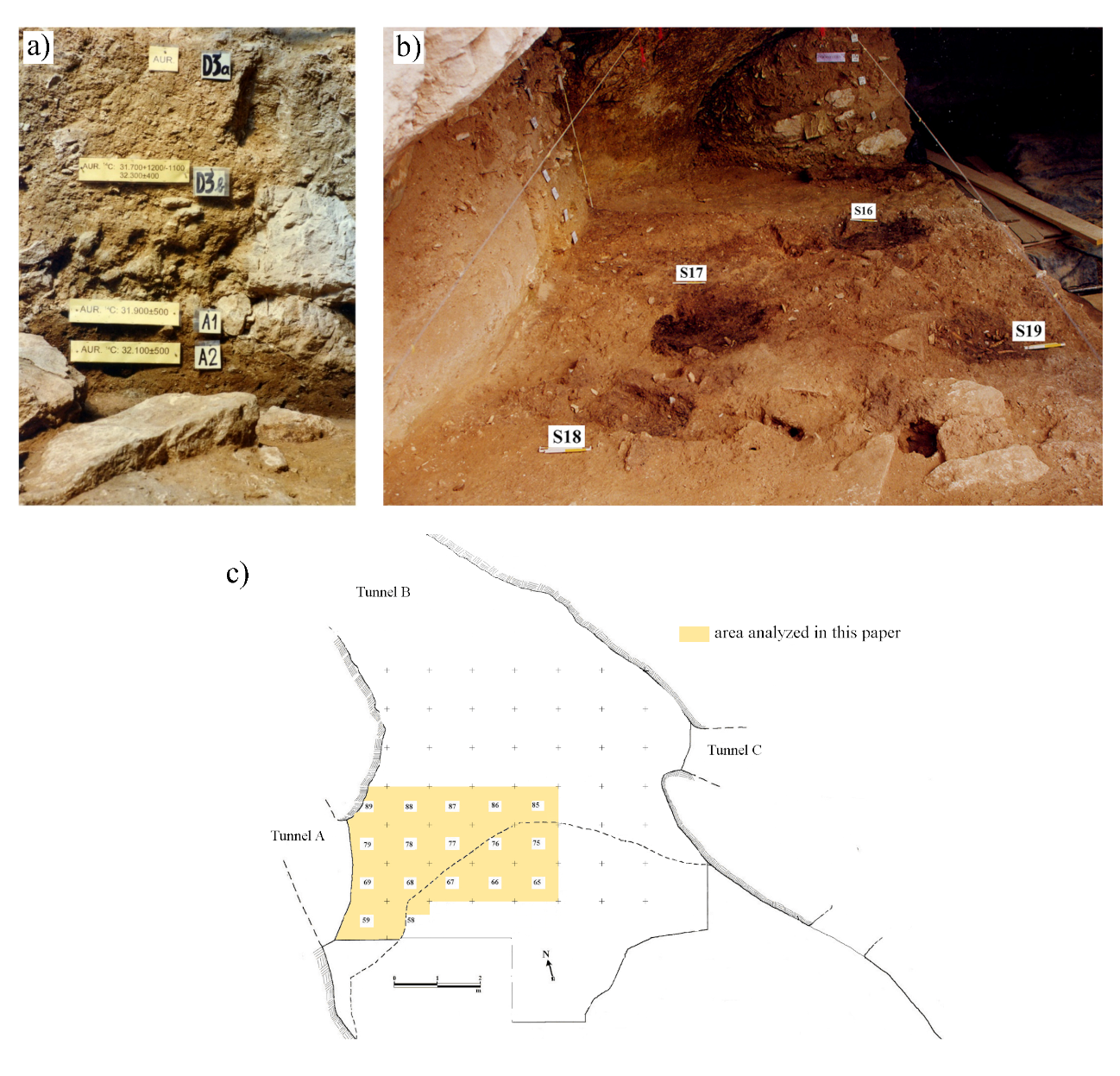


**Figure S2. a)** Stratigraphic profile at the cave entrance showing D3a, D3b, A1, and A2 units; **b)** combustion fea­tures, post-holes, and toss-zones in A2 unit (Photos: A. Broglio and M. Peresani); **c)** Cave entrance and the lateral tunnels showing the grid and excavation area. The area analysed in this paper is colored yellow. The present-day drip line is projected to the ground by the dotted line.


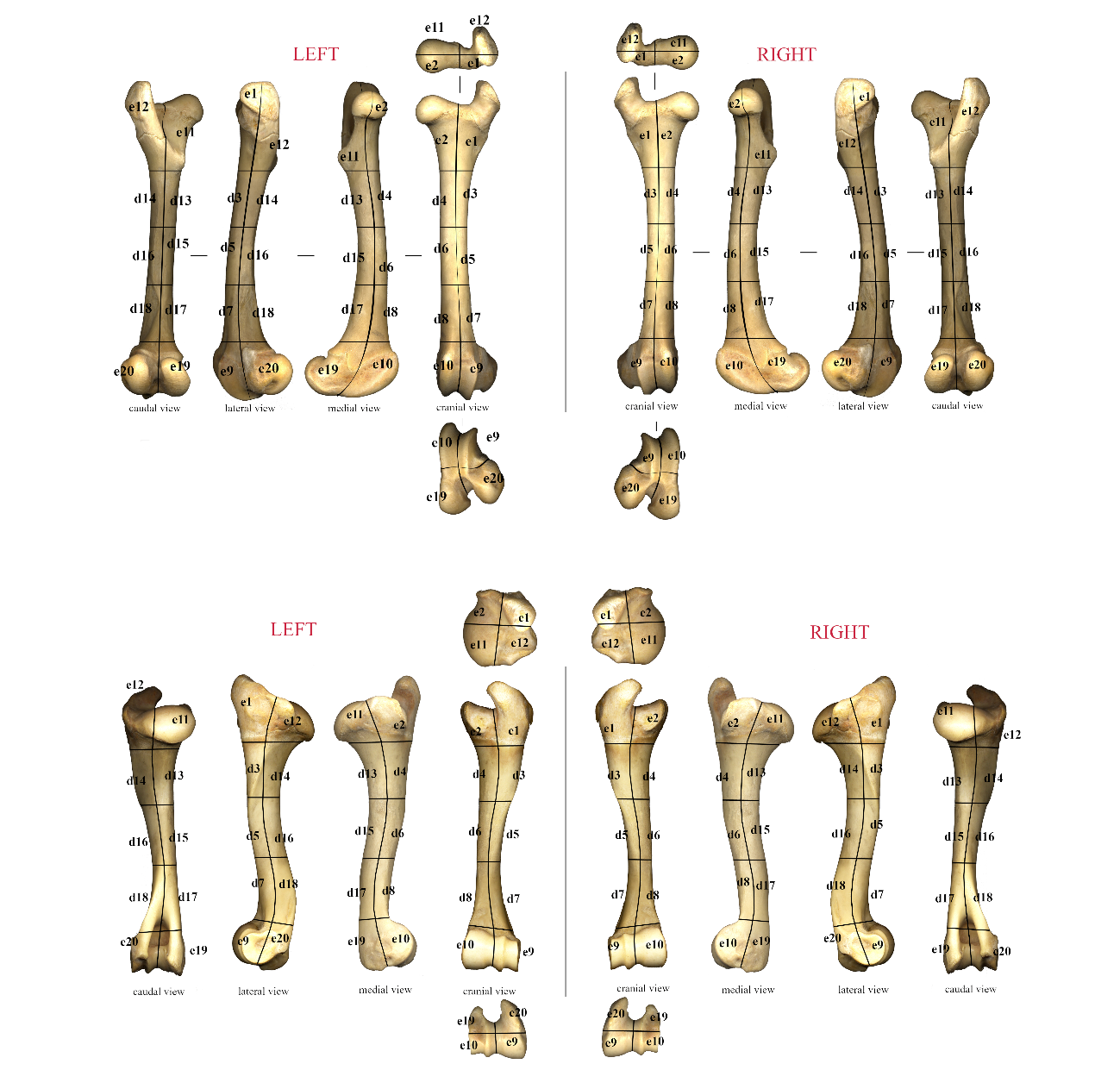
 **Figure S3.** Anatomical codes used for femur and humerus in this work to quantify the specific anatomical part of each bone.


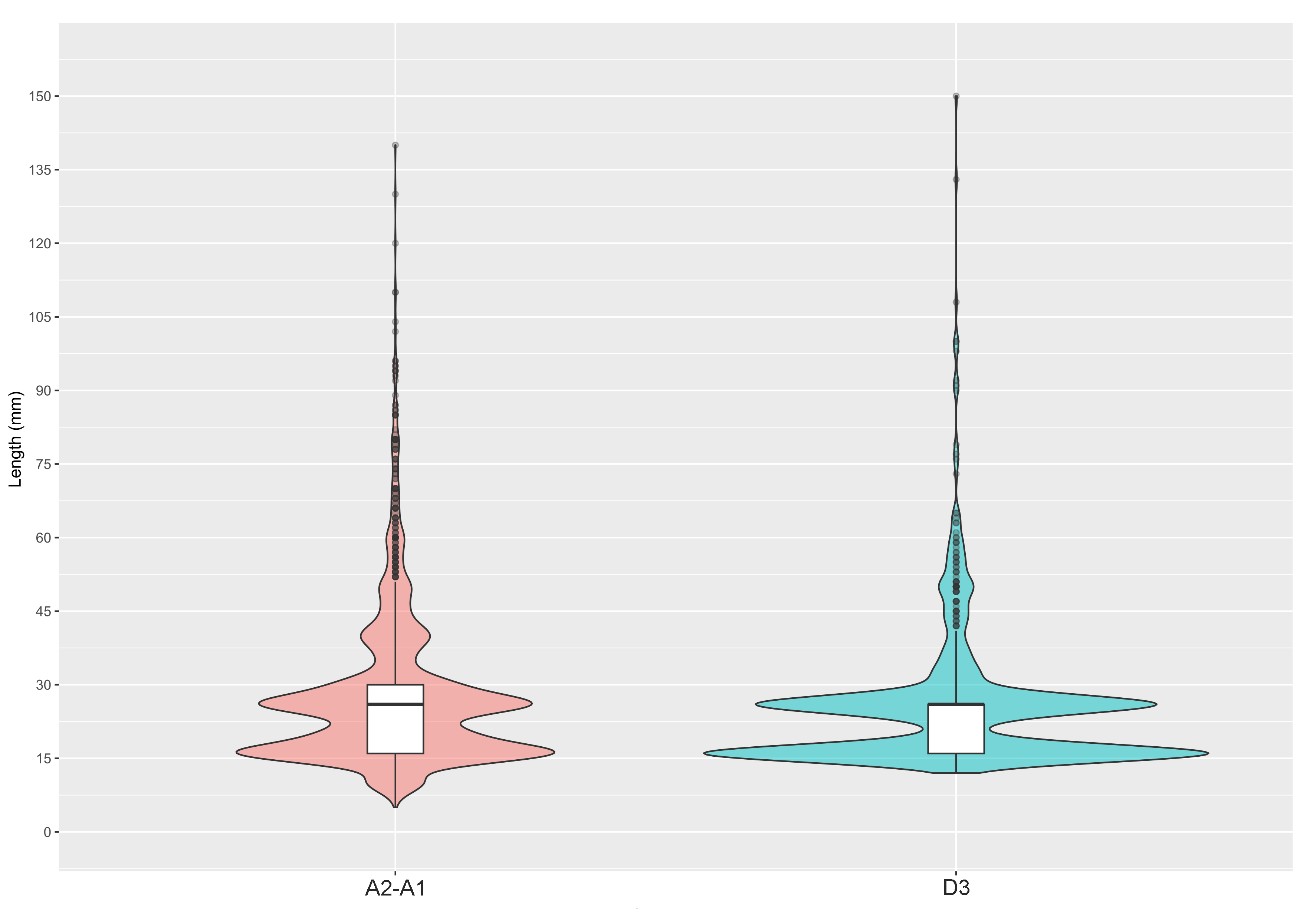


**Figure S4.** Length distribution of the bone assemblages found in the Protoaurignacian A2-A1 and D3 units


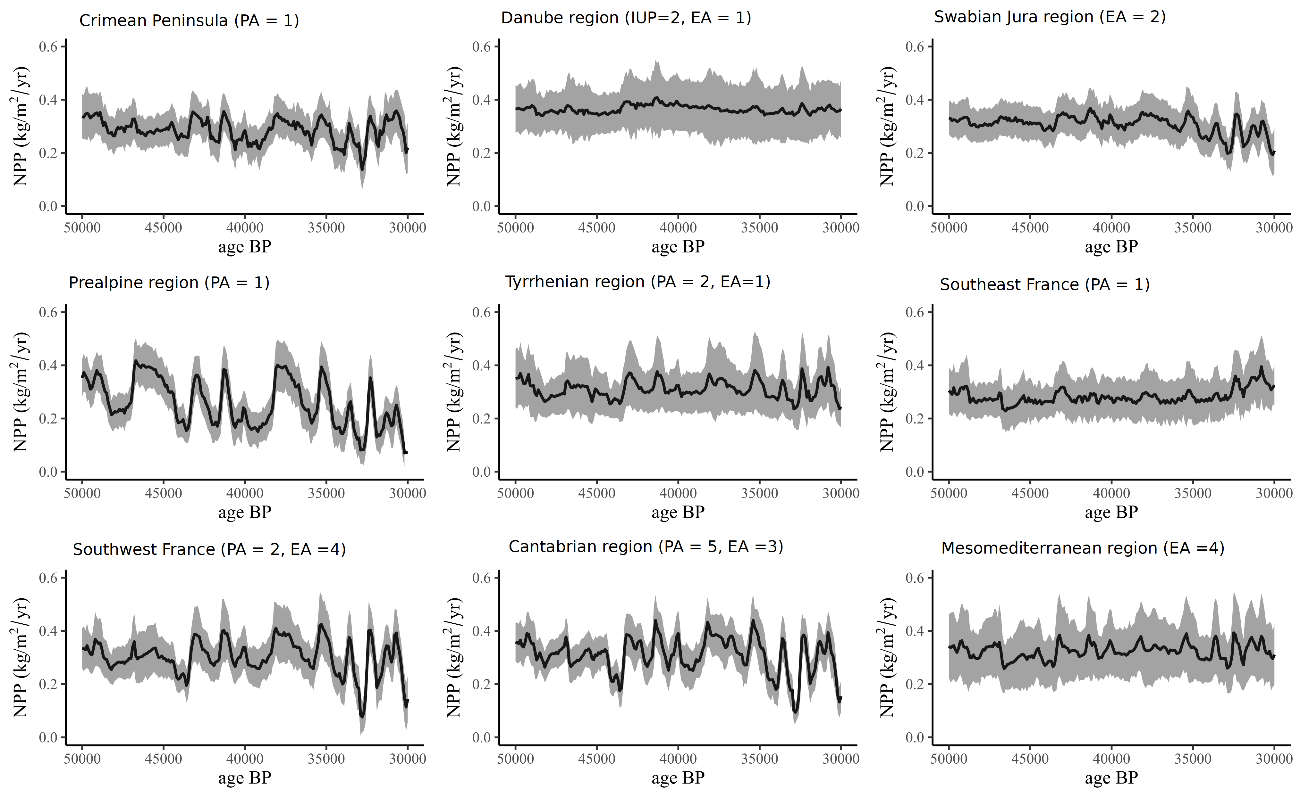


**Figure S5.** Temporal evolution of the mean Net Primary Productivity (NPP) shown by the black lines and the 95% CI in shaded grey in each biogeographic region during the late MIS 3. In parenthesis, the number of Proto (PA), Early Aurignacian (EA), and Initial Upper Palaeolithic (IUP) levels in each region.

**Code S1. CQL Individual Codes**

CQL Code of the model presented in Figure 1

Plot()

{

Outlier_Model("General",T(5),U(0,4),"t");

Sequence("Fumane Uluzzian and Protoaurignacian")

{

Boundary("A3");

Phase("Unit A3")

{

R_Date("OxA-X-2295-52", 41300, 1300)

{

Outlier("General", 0.05);

};

R_Date("OxA-21736", 39100, 1000)

{

Outlier("General", 0.05);

};

R_Date("OxA-41150", 38900, 1000)

{

color="red";

Outlier("General", 0.05);

};

R_Date("OxA-41151", 38080, 910)

{

color="red";

Outlier("General", 0.05);

};

Date("Uluzzian A3 unit");

};

Boundary("End UnitA3");

Phase("Unit A2-A1")

{

R_Date("OxA-41161", 36310, 750)

{

color="red";

Outlier("General", 0.05);

};

R_Date("OxA-19584", 35850, 310)

{

Outlier("General", 0.05);

};

R_Date("OxA-17569", 35640, 220)

{

Outlier("General", 0.05);

};

R_Date("OxA-21796", 35400, 750)

{

color="blue";

Outlier("General", 0.05);

};

R_Date("OxA-17570", 35180, 220)

{

Outlier("General", 0.05);

};

R_Date("OxA-19412", 34940, 280)

{

Outlier("General", 0.05);

};

R_Date("OxA-19414", 34180, 270)

{

Outlier("General", 0.05);

};

Date("Protoaurignacian A2+A1 unit");

};

Boundary("End Unit A2-A1");

Phase("Unit D3")

{

R_Date("OxA-41260", 36100, 1400)

{

color="red";

Outlier("General", 0.05);

};

R_Date("OxA-17981", 33890, 220)

{

Outlier("General", 0.05);

};

Date("Late Protoaurignacian D3 unit");

};

Boundary("End Unit D3");

};

};

**Code S2. CQL Individual Codes**

CQL Code of the model presented in Figure 5

(see Table S6 for further information)

Plot()

{

Outlier_Model("General",T(5),U(0,4),"t");

Sequence(Fumane)

{

Boundary("Unit A9");

Phase("Unit A9")

{

R_Date("LTL-376A", 42750, 700)

{

Outlier("General", 0.05);

};

R_Date("LTL-572A", 40150, 550)

{

Outlier("General", 0.05);

};

R_Date("OxA-11346", 39950, 550)

{

Outlier("General", 0.05);

};

Date("Duration Unit A9");

};

Boundary("End Unit A9");

Phase("Unit A5+A6")

{

R_Date("OxA-21757", 41500, 1500)

{

Outlier("General", 0.05);

};

R_Date("OxA-21758", 41100, 1300)

{

Outlier("General", 0.05);

};

R_Date("OxA-17566", 40460, 360)

{

Outlier("General", 0.05);

};

R_Date("OxA-21809", 40200, 1200)

{

Outlier("General", 0.05);

};

Date("Duration Unit A5+A6");

};

Boundary("End Unit A5+A6");

Phase("Unit A5")

{

R_Date("OxA-X-2275-45", 41650, 650)

{

Outlier("General", 0.05);

};

R_Date("OxA-17980", 40150, 350)

{

Outlier("General", 0.05);

};

R_Date("OxA-21712", 40000, 1100)

{

Outlier("General", 0.05);

};

Date("Duration Unit A5");

};

Boundary("End Unit A5");

Phase("Unit A4")

{

R_Date("OxA-21735", 42000, 1700)

{

Outlier("General", 0.05);

};

R_Date("OxA-21734", 42000, 1400)

{

Outlier("General", 0.05);

};

R_Date("OxA-21733", 41000, 1300)

{

Outlier("General", 0.05);

};

Date("Duration Unit A4");

};

Boundary("End Unit A4");

Phase("Unit A3")

{

R_Date("OxA-X-2295-52", 41300, 1300)

{

Outlier("General", 0.05);

};

R_Date("OxA-21736", 39100, 1000)

{

Outlier("General", 0.05);

};

R_Date("OxA-41150", 38900, 1000)

{

Outlier("General", 0.05);

};

R_Date("OxA-41151", 38080, 910)

{

Outlier("General", 0.05);

};

Date("Duration Unit A3");

};

Boundary("End Unit A3");

Phase("Unit A2-A1")

{

R_Date("OxA-41161", 36310, 750)

{

Outlier("General", 0.05);

};

R_Date("OxA-19584", 35850, 310)

{

Outlier("General", 0.05);

};

R_Date("OxA-17569", 35640, 220)

{

Outlier("General", 0.05);

};

R_Date("OxA-21796", 35400, 750)

{

Outlier("General", 0.05);

};

R_Date("OxA-17570", 35180, 220)

{

Outlier("General", 0.05);

};

R_Date("OxA-19412", 34940, 280)

{

Outlier("General", 0.05);

};

R_Date("OxA-19414", 34180, 270)

{

Outlier("General", 0.05);

};

Date("Duration Unit A2-A1");

};

Boundary("End Unit A2-A1");

Phase("Unit D3")

{

R_Date("OxA-41260", 36100, 1400)

{

Outlier("General", 0.05);

};

R_Date("OxA-17981", 33890, 220)

{

Outlier("General", 0.05);

};

Date("Duration Unit D3");

};

Boundary ("End Unit D3");

};
